# Genomic and Cytogenetic Analysis of Synthetic Polyploids between Diploid and Tetraploid Cotton (*Gossypium*) Species

**DOI:** 10.3390/plants12244184

**Published:** 2023-12-17

**Authors:** Mukhammad T. Khidirov, Dilrabo K. Ernazarova, Feruza U. Rafieva, Ziraatkhan A. Ernazarova, Abdulqahhor Kh. Toshpulatov, Ramziddin F. Umarov, Madina D. Kholova, Barno B. Oripova, Mukhlisa K. Kudratova, Bunyod M. Gapparov, Maftunakhan M. Khidirova, Doniyor J. Komilov, Ozod S. Turaev, Joshua A. Udall, John Z. Yu, Fakhriddin N. Kushanov

**Affiliations:** 1 Institute of Genetics and Plant Experimental Biology, Academy of Sciences of the Republic of Uzbekistan, Tashkent 111226, Uzbekistan; khidirov.tursunkilovich@gmail.com (M.T.K.); edilrabo64@gmail.com (D.K.E.); feruzarafiyeva25@gmail.com (F.U.R.); ziroat64@mail.ru (Z.A.E.); toshpolatovabduqahhor78@gmail.com (A.K.T.); ramziddinumarov96@gmail.com (R.F.U.); mxolova107@gmail.com (M.D.K.); barnoxonoripova93@gmail.com (B.B.O.); muhlisaqudratova216@gmail.com (M.K.K.); bunyodgapparov20@mail.com (B.M.G.); ozodturaev@gmail.com (O.S.T.); 2 Department of Genetics, National University of Uzbekistan, Tashkent 100174, Uzbekistan; khidirovamaftuna613@gmail.com; 3 Department of Biology, Namangan State University, Uychi Street-316, Namangan 160100, Uzbekistan; dkomilov81@mail.ru; 4 United States Department of Agriculture (USDA)-Agricultural Research Service (ARS), Southern Plains Agricultural Research Center, 2881 F&B Road, College Station, TX 77845, USA; joshua.udall@usda.gov

**Keywords:** cotton, *G. herbaceum*, *G. mustelinum*, interspecific hybridization, colchicine treatment, polyploidization, SSR markers, genomic changes

## Abstract

Cotton (*Gossypium* spp.) is the most important natural fiber source in the world. The genetic potential of cotton can be successfully and efficiently exploited by identifying and solving the complex fundamental problems of systematics, evolution, and phylogeny, based on interspecific hybridization of cotton. This study describes the results of interspecific hybridization of *G. herbaceum* L. (A_1_-genome) and *G. mustelinum* Miers ex Watt (AD_4_-genome) species, obtaining fertile hybrids through synthetic polyploidization of otherwise sterile triploid forms with colchicine (C_22_H_25_NO_6_) treatment. The fertile F_1_C hybrids were produced from five different cross combinations: (1) *G. herbaceum* subsp. *frutescens* × *G. mustelinum*; (2) *G. herbaceum* subsp. *pseudoarboreum* × *G. mustelinum*; (3) *G. herbaceum* subsp. *pseudoarboreum* f. *harga* × *G. mustelinum*; (4) *G. herbaceum* subsp. *africanum* × *G. mustelinum*; (5) *G. herbaceum* subsp. *euherbaceum* (variety A-833) *× G. mustelinum*. Cytogenetic analysis discovered normal conjugation of bivalent chromosomes in addition to univalent, open, and closed ring-shaped quadrivalent chromosomes at the stage of metaphase I in the F_1_C and F_2_C hybrids. The setting of hybrid bolls obtained as a result of these crosses ranged from 13.8–92.2%, the fertility of seeds in hybrid bolls from 9.7–16.3%, and the pollen viability rates from 36.6–63.8%. Two transgressive plants with long fiber of 35.1–37.0 mm and one plant with extra-long fiber of 39.1–41.0 mm were identified in the F_2_C progeny of *G. herbaceum* subsp. *frutescens* × *G. mustelinum* cross. Phylogenetic analysis with 72 SSR markers that detect genomic changes showed that tetraploid hybrids derived from the *G. herbaceum* × *G. mustelinum* were closer to the species *G. mustelinum*. The *G. herbaceum* subsp. *frutescens* was closer to the cultivated form, and its subsp. *africanum* was closer to the wild form. New knowledge of the interspecific hybridization and synthetic polyploidization was developed for understanding the genetic mechanisms of the evolution of tetraploid cotton during speciation. The synthetic polyploids of cotton obtained in this study would provide beneficial genes for developing new cotton varieties of the *G. hirsutum* species, with high-quality cotton fiber and strong tolerance to biotic or abiotic stress. In particular, the introduction of these polyploids to conventional and molecular breeding can serve as a bridge of transferring valuable genes related to high-quality fiber and stress tolerance from different cotton species to the new cultivars.

## 1. Introduction

Cotton belongs to the genus *Gossypium* which consists of approximately 46 diploid species and seven allotetraploid species [1,2,3]. All diploid (2n = 2x = 26) cotton species belong to eight genome groups (A-G and K), and all tetraploid (2n = 4x = 52) species are classified into one genome group (AD) [4,5]. *Gossypium* species spread through large geographical and ecological areas and has a wide range of morphological and genetic diversity, mainly preserved in germplasm collections and as genetic materials for cotton breeding programs worldwide. These resources can be successfully used to transfer valuable traits from wild species into elite cultivars [6,7]. Polyploidization is one mechanism that introduces the genetic diversity of diploids to polyploids, which allows easy crossing with tetraploids and obtaining fertile hybrids. Unfortunately, issues arise when diploid wild species are crossed with tetraploids. It is often impossible to obtain fertile hybrids through crossing between tetraploid and wild diploid species [8,9]. Completely sterile triploid (2n = 3x = 39) hybrids are obtained from the natural hybridization of tetraploid elite cultivars with wild diploid species [4]. Nevertheless, the fertility of hybrids can be recovered by polyploidization, resulting in hexaploid (2n = 6x = 78) plants [10]. 

The process of ancient genome duplication is called paleopolyploidy, which occurred at least several million years ago (MYA) [11]. In paleopolyploidy, the duplication of the genome of a single species is called autopolyploid, while combining the genomes of two species is allopolyploidy. Polyploidy is common in plants, fish, and some amphibians. Polyploids have more than two complete sets of chromosomes, which are inheritable. Diploid gametes can result in increased chromosome sets because of a failure to divide during meiosis. A triploid zygote is formed when these diploid gametes fuse with a monoploid gamete. These triploids can be unstable and sterile. When these diploid gametes fuse with another diploid gamete, a stable tetraploid zygote is formed. Polyploids can be tetraploid (4x), hexaploid (6x), and other aneuploids, higher order polyploids, or multiple chromosome pairs. The chromosome doubling through polyploidization can enhance the genetic diversity of plants.

The first comprehensive study in this field was conducted 250 years ago by Carl Linnaeus [12]. Polyploidization event was first discovered in 1907 and was considered responsible for increasing the number of chromosomes [13]. The development of plants that change as a result of polyploidization increases their adaptability to adverse conditions and environmental factors [14,15]. Currently, several chemical and gaseous agents are used in research to obtain polyploids. The most used agents are colchicine and oryzalin [16]. Colchicine (C_22_H_25_NO_6_) is obtained from the bulb-like corms of the plant *Colchicum autumnale* L. [17]. This substance is an alkaloid agent used to induce polyploidy [18]. This type of chemical binds to the tubulin protein and prevents the formation of microtubule spindle fibers (achromatin threads) during the metaphase of mitotic cell division, resulting in the duplication of chromosome bundles without spreading to the poles [19]. Colchicine can effectively stop cell division at the mitotic anaphase. At this stage, the chromosomes have already been duplicated, but mitosis has not yet occurred, and the restriction of cell wall formation at this stage leads to the formation of polyploid cells. They are usually larger than diploid species and often have thickening of the tissues, which leads to the formation of large plant organs [20]. 

The diversity of *Gossypium* species is an ideal model for understanding the evolution and domestication of polyploids, as well as the pathways and mechanisms of gene and genome evolution [21]. There are several large *Gossypium* germplasm collections around the world, and among the largest and richest germplasm collections are those in the USA and Uzbekistan. These germplasm collections consist of cotton accessions with high genetic diversity, and their widespread use in genetics and breeding is of great importance in solving the current problems facing the cotton industry [22,23]. 

The historical domestication and modern breeding of Upland cotton (*Gossypium hirsutum*) have significantly improved its yield and fiber quality, but this has led to a dramatically reduced genetic diversity [24,25,26,27]. Upland cotton cultivars have a narrow genetic base of fiber quality and abiotic stress tolerance traits. The gene introgression from species such as *G. herbaceum* and *G. mustelinum* allows the increase of genetic diversity by introducing new alleles to improve fiber quality and stress tolerance. *G. herbaceum*, also known as ‘Levant cotton’, is grown in rainfed areas of Africa, and this crop is tolerant to salinity, drought, and scorching hot weather [28]. *G. mustelinum* is a wild species native to the northeastern region of Brazil, among existing allotetraploids [29]. The fiber of *G. mustelinum* is genetically different from that of allotetraploid *G. hirsutum*. The quantitative trait loci (QTL) for fiber quality and the interesting alleles identified from *G. mustelinum* can make an important contribution to the improvement of Upland cotton germplasm [25]. Wang et al. [30] dissected the molecular genetic basis of fiber strength and fineness in *G. mustelinum* and *G. hirsutum* crosses. They identified 42 QTLs, highlighting the potential of *G. mustelinum* alleles to improve fiber quality in Upland cotton. The study revealed new allelic variation for cotton breeding. Yang et al. [31] made a chromosome-level genome assembly of *G. mustelinum*, demonstrating its efficacy in identifying genes for qualitative and quantitative traits. The study laid a foundation for cotton genetics and breeding, emphasizing the rich gene pool of *G. mustelinum*. Chen et al. [25] used selective genotyping to validate over 75 QTLs for fiber quality traits that were introgressed from *G. mustelinum* into Upland cotton. The study lays the foundation for fine mapping, marker-assisted selection, and map-based gene cloning.

In the past few decades, the following studies were conducted on obtaining interspecific hybrids exploiting the genetic diversity of *Gossypium* germplasm: *G. hirsutum × G. klotzschianum* [32], *G. hirsutum × G. trilobum* [33], *G. arboreum × G. anomalum* [34], *G. hirsutum × G. anomalum* [35], *G. herbaceum × G. australe* [36], *G. hirsutum × G. australe* [37], *G. hirsutum × G. arboreum* [38], *G. capitis-viridis* × (*G. hirsutum × G. australe*)^2^ [39], *G. hirsutum × G. darwinii* [40], *G. herbaceum × G. nelsonii* [41]. Despite an extensive literature review, no reports were found on obtaining allotetraploids using the cotton species *G. herbaceum* and *G. mustelinum*. 

In this study, we hypothesized that obtaining fertile hybrids through the polyploidization of triploids derived from the cross between *G. herbaceum* and *G. mustelinum* could enable their use in tetraploid cotton breeding. To test this hypothesis, the goals of our research were as follows: (1) to obtain F_1_ triploid hybrids of *G. herbaceum* and *G. mustelinum* cotton species; (2) to obtain F_1_C hexaploids through doubling the genome of triploid hybrids using colchicine treatment; and (3) to select tetraploids among segregating F_2_C aneuploid (triploid, tetraploid, and hexaploid) hybrids. The new knowledge and genetic resources would be used in the introduction of valuable traits such as fiber quality and stress tolerance into the elite Upland cotton varieties using marker-assisted selection (MAS) technology.

## 2. Results

### 2.1. The Characteristics of Interspecific Cotton Hybrids between G. herbaceum and G. mustelinum 

Several crosses were made between *G. herbaceum* (A_1_ genome) and *G. mustelinum* (AD_4_ genome) species to develop interspecific hybrids with valuable traits as initial materials for genetics and breeding studies. As a result of many crossings, the interspecific triploid F_1_ hybrids were obtained in five different combinations such as (1) *G. herbaceum* subsp. *frutescens* × *G. mustelinum*, (2) *G. herbaceum* subsp. *pseudoarboreum* × *G. mustelinum,* (3) *G. herbaceum* subsp. *pseudoarboreum* f. *harga* × *G. mustelinum*, (4) *G. herbaceum* subsp. *africanum* × *G. mustelinum,* and (5) *G. herbaceum* subsp. *euherbaceum* (variety A-833) × *G. mustelinum*. The boll-setting rate of the hybrids in the recipient buds after crossing was 13.8–92.2%, and complete seed-setting rate in hybrid bolls was 9.7–16.3%. The highest rate of fertility was observed between variety A-833 (subsp. *euherbaceum*) and subsp. *frutescens* (cultivated tropical form), and boll-setting rate was over 50.0%. A relatively low fertility rate was recorded in subsp. *pseudoarboreum* × *G. mustelinum* (13.8%). Complete seed-setting rates showed relatively similar among all combinations (9.7–16.3%) (Table 1).

**Figure 1:**
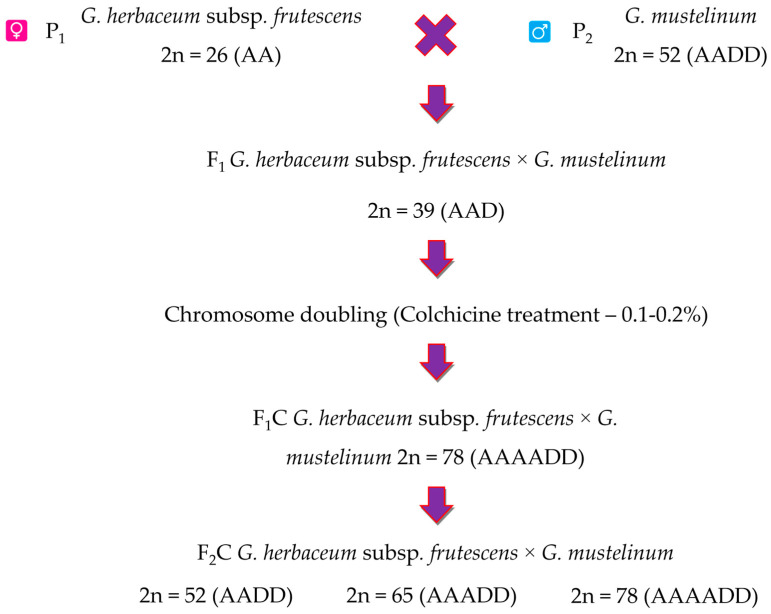
The scheme of obtaining cotton amphidiploids.

Typically, triploids are infertile, some of them are semi-fertile and can produce both aneuploid and euploid gametes. Taking this into account, the resulting F_1_ triploid hybrid seeds were treated with colchicine before sowing. Triploid and polyploid hybrids were obtained as following scheme (Figure 1).

**Figure 2:**
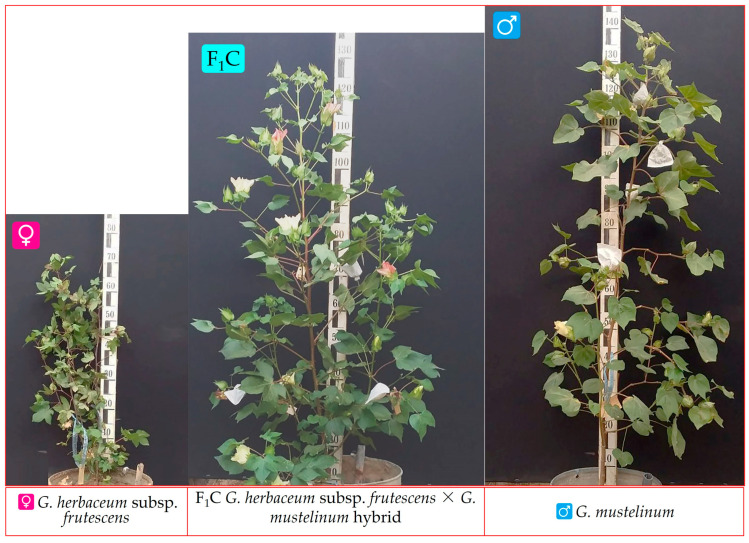
(♀)—diploid *G. herbaceum* subsp. *frutescens*; (♂)—tetraploid *G. mustelinum*; F_1_C—allohexaploid hybrid progeny of the first generation of *G. herbaceum* subsp. *frutescens* × *G. mustelinum*.

Only, *G. herbaceum* subsp. *africanum* × *G. mustelinum*, subsp. *pseudoarboreum* f. *harga* × *G. mustelinum*, subsp. *frutescens* × *G. mustelinum* (Figure 2) combinations were grown after colchicine treatment to produce viable F_1_C hybrids.

### 2.2. Inheritance of Morphological and Economically Valuable Traits in Interspecific Polyploid Hybrids F_1_C and F_2_C

#### 2.2.1. The Number of Bolls per Plant and the Fertility of Seeds

It is known that parameters such as the number of bolls in a plant, fiber yield, weight of 1000 seeds, weight of cotton in one boll and the number of fully born seeds in one boll play an important role in determining the level of productivity. Since these indicators are inextricably linked with each other and have a correlative effect on the development of the character. In addition, qualitative and quantitative indicators of seeds born in one bag are used as a basis for determining the phylogenetic relationships of species based on classical methods. Therefore, the initial sources used in the rese—arch and the yield indicators of the polyploid hybrids obtained on their basis were analyzed. In terms of the number of bolls per plant among the subspecies of *G. herbaceum*, the highest rate (36.0 pieces) was recorded in the form of subsp. *pseudoarboreum* f. *harga*. In the analyzed bolls, the rate of complete seed (or, fertility of seeds) was 89.2 ± 1.9%. Also, the number of bolls per plant showed positive indicators on subspecies of subsp. *africanum*—27.0 pieces, and the yield of complete seeds in the boll was 94.5 ± 1.8%, in the variety A-833 (subsp. *euherbaceum*)—28.0 pieces, and the rate of complete seeds was 86.9 ± 1.9%. In *G. mustelinum*—a Brazilian endemic species, the average number of bolls per plant was—16.0, and the rate of complete seed was—86.0 ± 2.1% (Table 2).

In F_1_C subsp. *frutescens* × *G. mustelinum* polyploid hybrids, the number of bolls per plant was—21.0, and the “fertility rate of seeds per bolls” was very low (19.4 ± 1.9%) (Table 3). An average of 14.3 seeds were set in one boll, of which 2.8 whole seeds and 11.5 empty seeds were set. In F_1_C subsp. *pseudoarboreum* f. *harga* × *G. mustelinum* hybrids, the number of bolls per plant was—1.0, the number of complete seeds was 2.0, and the number of empty seeds was 7.0. F_1_C subsp. *africanum* × *G. mustelinum* hybrid plants were sterile, and no yield components were observed.

In hybrid plants of subsp. *frutescens* × *G. mustelinum* F_2_C generation, there was a variation of 18.0–26.0 units in terms of “number of bolls per plant”. Relatively low values (between 30.6 ± 1.2% and 49.2 ± 2.1%) were found in the “seeds fertility” indicator, also. According to the results of the analysis, in polyploid hybrids of the F_1_C and F_2_C generations, there was a slight decrease in the number of pods per plant compared to the parental samples, as well as cases of sterility in the subsp. *africanum* × *G. mustelinum* combination. A sharp decrease was noted in terms of the rate of complete seed fertility, which is the main factor of productivity. This indicates that the parental lines are phylogenetically distant and isolated species.

#### 2.2.2. Vegetative Growth Duration

According to the results of the research, wild (subsp. *africanum*) and ruderal (subsp. *pseudoarboreum* f. *harga*) forms of *G. herbaceum* of which, the vegetative growth duration was 137.4 and 127.2 days, therefore they would require breeding for short day conditions. The vegetative growth duration in the cultivated-tropical subsp. *frutescens*, ruderal subsp. *pseudoarboreum* subspecies and cultivated subsp. *euherbaceum* A-833 variety is 117.2, 119.6, and 117.4 days respectively, in field conditions since they do not require breeding for short days. The wild tetraploid species *G. mustelinum* also requires breeding for short days since the duration of its vegetative growth duration was 151.8 days.

F_1_C hybrid plants obtained by cross-species with representatives of the diploid *G. herbaceum* species and tetraploid *G. mustelinum* species was between 128.6–145.2 days. Including F_1_C subsp. *frutescens* × *G. mustelinum* the vegetative duration of polyploid hybrid plants was 128.6 days, the coefficient of variation was 2.3%. The coefficient of dominance for the sign is equal to *hp* = 0.34, and the state of partial dominance of the form with a positive indicator (subsp. *frutescens)* was noted. The vegetative growth duration of F_1_C subsp. *pseudoarboreum* f. *harga* × *G. mustelinum* polyploid hybrid combination plants was 139.6 days on average, the variation coefficient was 1.5%, and the dominance coefficient was *hp* = −0.01. In this combination, the character was inherited with a partial dominance of the negative indicator form (*G. mustelinum*). The average vegetative growth duration of polyploid hybrid plants was 139.6 days, the variation coefficient was 1.5%, and the dominance coefficient was *hp* = −0.01. In this combination, the character was inherited in the partial dominance of the *G. mustelinum* species with a negative indicator. The polyploid hybrid plants of F_1_C subsp. *africanum* × *G. mustelinum* combination had an average vegetative growth duration of 145.2 days, and the coefficient of variation was 2.2% (Table 4). The coefficient of dominance for the studied character is equal to *hp* = −0.08, and in this combination, the character was inherited in the case of partial dominance of the negative indicator form (*G. mustelinum*).

From the above-mentioned F_1_C polyploid hybrids, only F_2_C hybrids of subsp. *frutescens* × *G. mustelinum* combination were obtained. Also, in this generation, the degree of variability of the indicators of the period from the germination to the opening of the first bud was studied in 50% of the plants. A total of 134 plants were obtained in the F_2_C generation of the subsp. *frutescens* × *G. mustelinum* combination, of which 53 strongly required breeding for short days and thus affected yield components under field conditions. In the remaining 81 F_2_C hybrids, the growth duration showed a wide range of variability between 116.6–152.2 days, which were divided into eight classes. Among the studied hybrid combinations, the growth duration was 115–119 day and faster recombinant forms around 120–124 were isolated.

#### 2.2.3. Fiber Length and Strength

Cotton has been cultivated for centuries mainly for its fiber and has undergone natural and artificial selection. The wild diploid and tetraploid species are the natural resources with high genetic diversity for improving the fiber quality traits of elite cultivars. Therefore, fiber length is considered one of the major indicators of valuable economic traits in the breeding process.

According to the results of fiber quality tests the fiber length was in the range of 17.2–25.2 mm in *G. herbaceum* subspecies (Table 5). In this case, a relatively low result was observed in subsp. *frutescens* (17.2 ± 0.2 mm) and a relatively high result was observed in cultivated variety subsp. *euherbaceum* A-833 (25.2 ± 0.3 mm). As well as 25.1 ± 0.4 mm was in ruderal subsp. *pseudoarboreum*, 20.1 ± 0.2 mm was in subsp. *pseudoarboreum* f. *harga* and 24.7 ± 0.3 mm was in wild-type subsp. *africanum*. In the case of tetraploid *G. mustelinum*, the fiber length was 25.6 ± 0.2 mm.

The fiber strength varied in all of the cotton samples including the highest values of 35.5 cN/tex and 32.6 cN/tex, respectively, in subsp. *frutescens* and subsp. *africanum*. The fiber strength in A-833 variety of subsp. *euherbaceum* was 27.2 cN/tex, in subsp. *pseudoarbareum* f. *harga* was 24.1 cN/tex, and in *G. mustelinum* was 17.3 cN/tex (Table 5). 

Due to two F_1_C allohexaploid combinations were dead after the germination and the other two being male sterile, therefore all analyses were conducted only with *G. herbaceum* subsp. *frutescens* × *G. mustelinum* F_1_C allohexaploids since they were fertile. The fiber length was 28.7 ± 0.9 mm, and a positive heterosis (hp = 1.74) was observed. 

Variability was observed in the plants of F_2_C subsp. *frutescens* × *G. mustelinum* hybrid combinations. The analyzed results were divided into eight classes. Accordingly, two transgressive plants with long fiber of 35.1–37.0 mm, and one transgressive plant with extra-long fiber of 39.1–41.0 mm were identified. Since the fiber of the Brazilian endemic species *G. mustelinum* differs from that of the allotetraploid species *G. hirsutum* in terms of strength, maturity and length, a genetically rich source for improving fiber qualities of cultivars. 

The identification of major QTL loci for fiber quality and application of a set of beneficial alleles from *G. mustelinum* can contribute significantly to the long-term improvement of cultivated cotton germplasm. To introduce important alleles from *G. mustelinum* species into the genome of *G. hirsutum* species for fiber quality improvement, interspecific populations consisting of plants obtained by crossing *G. mustelinum* species with *G. hirsutum* species were created [43,44,45]. In the studied families, it was determined that the genes responsible for fiber strength and fineness in alleles of *G. mustelinum* are dominantly inherited. Based on the previous work with *G. barbadense*, *G. tomentosum*, and *G. darwinii*, which included introgression of *G. mustelinum* alleles [43,44,45]. These hybrid genotypes identified in our study would serve as a valuable source for the development of long-fiber cultivars and future breeding programs.

### 2.3. Cytogenetic and Genomic Studies of Interspecific Hybrids

Chromosome conjugation, spore and pollen viability analysis at metaphase I (MI) stage of meiosis is one of the effective methods for determining the ploidy level of intergenomic hybrids (allopolyploids, autopolyploids). Cytogenetic analyzes of hybrids resulting from the crosses between diploid and tetraploid cotton species were conducted in the study, and interesting data were obtained regarding their genomic structure and their partial homology.

**Figure 3:**
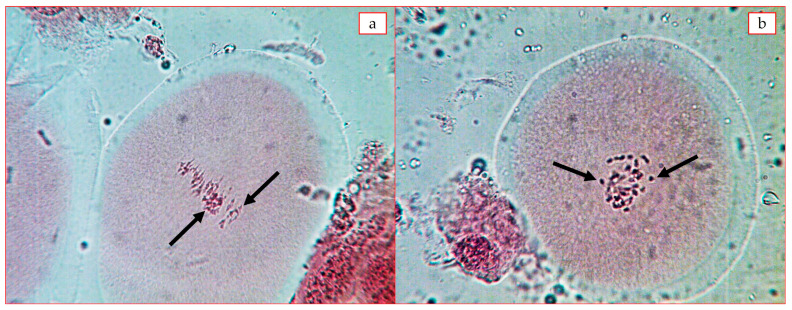
Conjugation of chromosomes at metaphase-I stage of meiosis: (**a**) F_1_C *G. herbaceum* subsp. *frutescens* × *G. mustelinum* 35^II^ + 2^IV^; (**b**) F_1_C *G. herbaceum* subsp. *pseudoarboreum* f. *harga* × *G. mustelinum* 38^II^ + 2^I^ (arrows indicate quadrivalents and univalents).

In the three hexaploid plants studied, disturbances in the process of the first division of meiosis (MI) were detected. F_1_C subsp. *frutescens* × *G. mustelinum* 38.22 ± 0.25 bivalents, 0.45 ± 0.29 univalents (the number of univalents was up to six) and 0.36 ± 0.14 quadrivalents. F_1_C subsp. *pseudoarboreum* f. *harga* × *G. mustelinum* and F_1_C subsp. *africanum* × *G. mustelinum* combinations had a higher number of univalents than the above combination (up to 2–10) (Table 6, Figure 3). The analysis showed that due to structural differences in chromosomes and the impossibility of normal chromosome conjugation in hybrids, there was a desynaptic effect due to early and asynchronous divergence in individual bivalents (Figure 3 a,b).

In general, the number of univalents found in maternal pollen cells was recorded from two to ten. It was known that there is weak desynapsis (meeting of several univalents along with bivalents in the female pollen cell), medium desynapsis (meeting of many univalents along with bivalents in the female pollen cell), and complete desynapsis (meeting of mainly univalents and sometimes several bivalents in the female pollen cell) [46]. Such univalents were formed as a result of early divergence of chromosomes. In the cotton samples of our study, this level—from two to ten univalent encounters—corresponded to the medium desynapsis.

**Figure 4:**
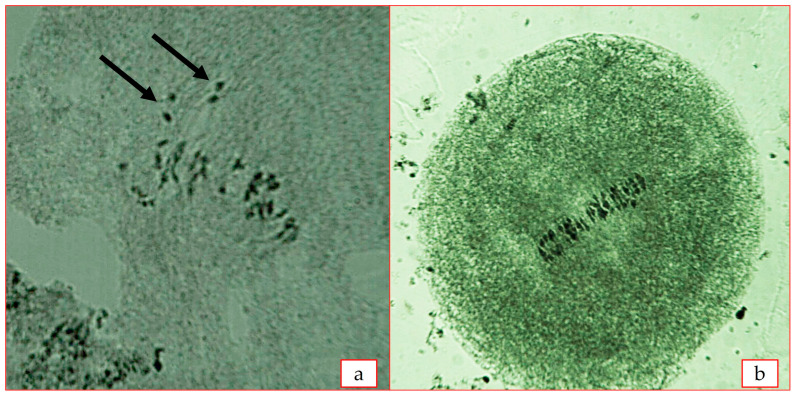
Conjugation of chromosomes at metaphase-I stage of meiosis—F_2_C *G. herbaceum* subsp. *frutescens* × *G. mustelinum* (**a**)—24^II^ + 4^I^ (arrows indicate univalents); (**b**)—26^II^.

As a result of research, the MI phase of meiosis was observed in some plants of the F_2_C subsp. *frutescens* × *G. mustelinum* hexaploid hybrid (Figure 4). The cotton plant samples with different levels of ploidy (24.73^II^; 38.18^II^; 38.00^II^) were identified in second generation (F_2_C). Disruptions in the mentioned chromosomal conjugation led to partial sterility of anthers in hexaploid hybrids and abnormal spore formation at the sporulation stage. It should also be noted that the MI phase of meiosis was carried out normally (26.00^II^) in two hybrid plants (Table 6).

Thus, the cause of partial infertility observed in allopolyploids was likely due to the presence of disturbances in the meiosis phase (disruption of the synchronous distribution of chromosomes in the anaphase or desynapsis phenomenon), instability of the number of chromosomes. One of the unique features of hybrids derived from the crosses between cotton species far from each other was with chromosomal aberrations. In such cases, plants of with chromosomal aberrations are due to the absence of homologous chromosomes of different species or the presence of their only partial homologue. Hybrids with homeologous genomes are viable, but sterile, i.e., sterile, due to genome duplication [47]. According to the conclusions of Sanamyan [48], hybrid combinations obtained from the diploid species (*G. thurberi* × *G. raimondii*, *G. arboreum* × *G. thurberi*, and *G. herbaceum* × *G. thurberi*) are productive in generations, on the contrary, intergenomic hybrids are very a large percentage of plants without flowers to appear.

There were difficulties in spore analysis to determine the required stage due to the very low number of spikelets and pollen grains in polyploid hybrids. As a result, ten additional samples had to be analyzed. According to the analysis of the conducted tetrads, 90.3–96.8% meiotic stage in three hybrid forms (F_1_C subsp. *frutescens* × *G. mustelinum*, F_1_C subsp. *pseudoarboreum* f. *harga* × *G. mustelinum*, F_1_C subsp. *africanum* × *G. mustelinum*) index was defined. It was noted that certain disturbances of the meiotic index in the form of micronuclear tetrads and polyads were observed in all forms. In F_1_C subsp. *frutescens* × *G. mustelinum* polyploid hybrid, the meiotic index was 90.36%, the rate of micronuclear tetrads was 3.27% and that of polyads was 6.37% (Table 7).

Micronuclear tetrads were not found in F_1_C subsp. *pseudoarboreum* f. *harga* × *G. mustelinum* polyploid hybrid unlike other plants. F_2_C subsp. *frutescens* × *G. mustelinum* polyploid hybrids T 38-12, T 30-6, T 30-7, T 30-8, T 38-3 forms meiotic index 96.05–98.54%, polyads 1.42–3.12%, micronuclear tetrads were not observed. In T 1-8 and T 51-13 forms, meiotic index was 96.15–97.45%, polyads 2.11–3.07%, micronuclear tetrads 0.69–0.89%. 

**Figure 5:**
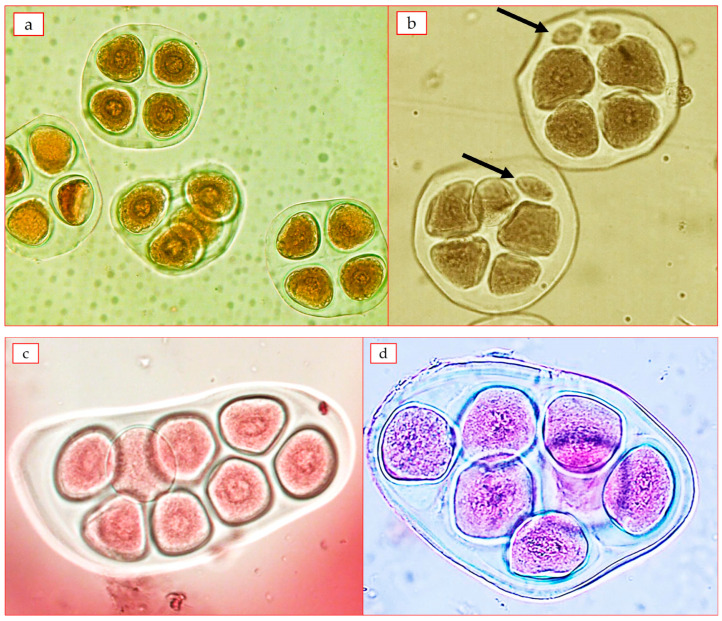
Microscopic images of tetrads in the example of F_1_C *G. herbaceum* subsp. *frutescens* × *G. mustelinum* hybrid: (**a**)—normal tetrads; (**b**)—micronuclear tetrads (arrows indicate micronuclei); (**c**)—octad; (**d**)—heptad (at 40× magnification).

In tetrads with identified micronuclei, up to 1–8 micronuclei (Figure 5c), and from polyads up to pentad, hexad, heptad, octad (Figure 5d) aneuploid spores were noted.

**Figure 6:**
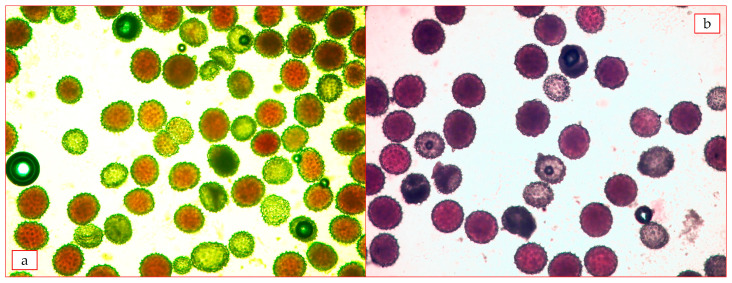
The results of pollen fertility analysis: (**a**)—F_1_C *G. herbaceum* subsp. *pseudoarboreum* f. *harga* × *G. mustelinum* 63.89 ± 3.55; (**b**)—F_1_C *G. herbaceum* subsp. *frutescens* × *G. mustelinum* 32.67 ± 3.55 (at 40× magnification).

It was known that pollen grains formed as result of meiosis are not equal in terms of genetic characters and functional capabilities. When the chromosomal complex of pollen grains was insufficient (i.e., when the frequency of meeting homologous chromosomes was low, the pollen grains were underdeveloped, and the viable pollen decreased significantly). When a complete chromosome complex was obtained, that is, when the homologous row of chromosomes was restored, the pollen grains became pink and fertile again. As a result of cytogenetic analysis, pollen graininess in hybrid plants F_1_C subsp. *pseudoarboreum* f. *harga* × *G. mustelinum* 63.89% in the combination of, F_1_C subsp. *frutescens* × *G. mustelinum* combination had a low yield—32.67%, F_1_C subsp. *africanum* × *G. mustelinum* combination, pollen sterility was determined (Table 8, Figure 6).

Since most of the hybrid plants showed short day and late tolerance in F_2_C generation of the combination subsp. *frutescens × G. mustelinum*, 24 out of 134 plants were studied for pollen graininess (Table 9). Until late autumn, reproductive organs were not formed in many hybrids, abnormal forms were noted without pollen development in hybrids that started flowering. There were a few cases where dust grains were not formed.

**Figure 7:**
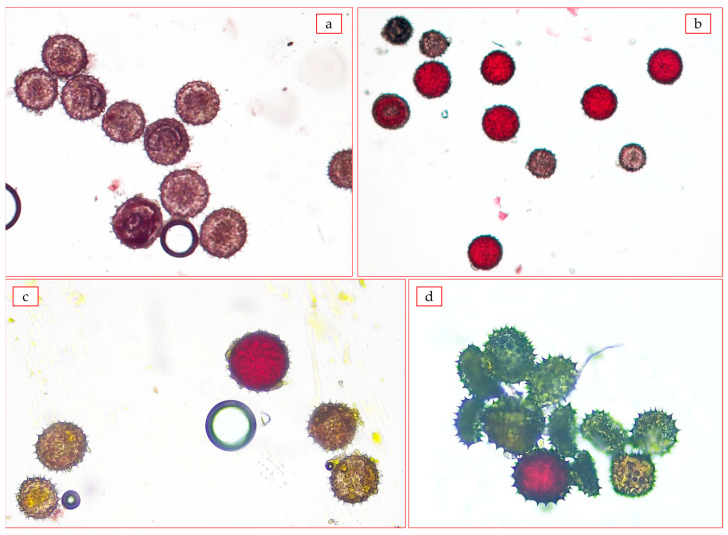
The results of pollen fertility analysis (F_2_C *G. herbaceum* subsp. *frutescens* × *G. mustelinum*): (**a**) T 1-4—40.43%; (**b**) T 37-4—50.2%; (**c**) T 49-2—1.5%; (**d**) T 37-14—7.9%.

The pollen fertility in the studied hybrids had different indicators. The pollen fertility of 14 hybrids was low (1.49–40.43%). Among them T 33-6, T 30-8, T49-2, T 38-7 plants were very low (1.49–10.23%), T 33-2, T 37-4, plants up to 50% or more, T 38-3, T 30-6, T 30-7, T 38-12, more than 60% (Table 8, Figure 7) indicators of dustiness were found in plants.

### 2.4. Molecular and Phylogenetic Analysis of Allopolyploid Forms of Cotton

**Figure 8:**
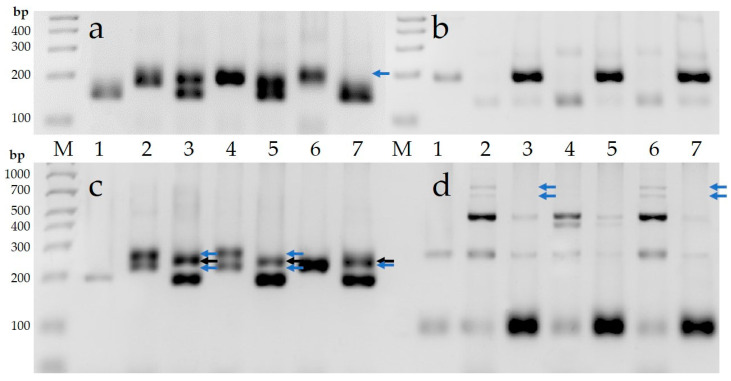
Genetic polymorphisms and genomic changes of the cotton samples using SSR markers. (**a**) NAU1458 marker; (**b**) NAU1093 marker; (**c**) BNL2634 marker; (**d**) BNL3140 marker. M—Molecular weight marker (base pairs, bp), 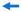
—lack alleles, 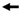
—additional alleles. 1—*G. mustelinum*; 2—*G. herbaceum* subsp. *frutescens*; 3—F_1_C (*G. herbaceum* subsp. *frutescens* × *G. mustelinum*); 4—*G. herbaceum* subsp. *pseudoarboreum* f. *harga*; 5—F_1_C (*G. herbaceum* subsp. *pseudoarboreum* f. *harga* × *G. mustelinum*); 6—*G. herbaceum* subsp. *africanum*; 7—F_1_C (*G. herbaceum* subsp. *africanum* × *G. mustelinum*).

The PCR analysis was conducted using 72 simple sequence repeat (SSR) markers (Table S1) associated with economically important traits to determine the genetic polymorphisms between parental genotypes as well as to determine some genomic changes in allohexaploid hybrids (Figure 8). As a result of PCR analysis, 57 DNA markers were determined as polymorphic markers (Table S2), while 14 DNA markers were monomorphic, and one DNA marker was not amplified. 

Based on the results of PCR analysis, the genomic changes were determined in the genomic regions of 18 (31.6%) out of 57 polymorphic DNA markers or in 40 (26.7%) PCR amplicons (alleles) of 150 alleles in allohexaploid hybrids. In particular, 31 (26.0%), 29 (24.3%), and 27 (22.6%) alleles had a change in three F_1_C combinations (*G. herbaceum* subsp. *frutescens* × *G. mustelinum*), F_1_C (*G. herbaceum* subsp. *pseudoarboreum* f. *harga* × *G. mustelinum*) and F_1_C (*G. herbaceum* subsp. *africanum* × *G. mustelinum*) hybrids, of 119, 120 and 116 alleles, respectively. In these 3 allohexaploid hybrids, alleles 23, 21, and 20 were missing although they were present in parental genotypes, respectively. On the other hand, non-parental alleles 8, 8, and 7 appeared in allohexaploid hybrids, respectively.

**Figure 9:**
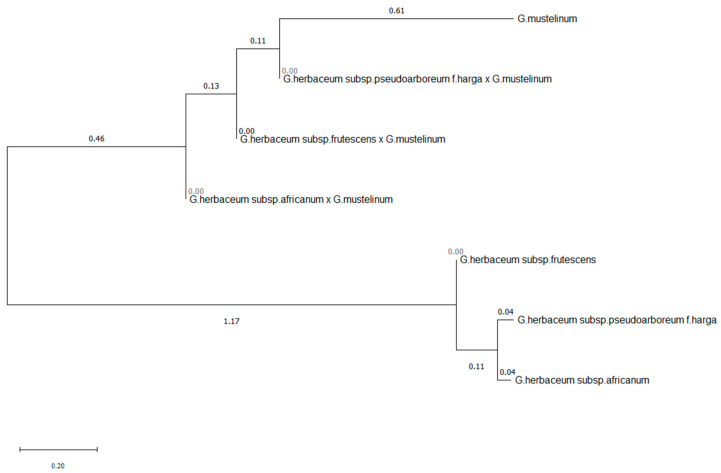
The phylogenetic tree of cotton species and their interspecific alloploid hybrids using 57 polymorphic SSR markers. With the phylogenetic genealogy method, the arithmetic values of the genetic regions of the cotton species and their interspecific polyploid hybrids were identified using the Neighbor-Joining method of the PAUP 4.0 (Phylogenetic Analysis Using Parsimony and other methods) program.

A phylogenetic tree construction indicated that the cotton accessions were clustered into two main groups (Figure 9). The first group included three *Gossypium herbaceum* subspecies such as subsp. *frutescens*, subsp. *pseudoarboreum* f. *harga* and subsp. *africanum*. *G. herbaceum* subsp. *pseudoarboreum* f. *harga* and subsp. *africanum* are genetically more similar to each other than subsp. *frutescens*. *G. mustelinum* and three interspecific allohexaploid hybrids F_1_C (*G. herbaceum* subsp. *frutescens* × *G. mustelinum*), F_1_C (*G. herbaceum* subsp. *pseudoarboreum* f. *harga* × *G. mustelinum*) and F_1_C (*G. herbaceum* subsp. *africanum* × *G. mustelinum*) were presented in the second group of the phylogenetic tree.

## 3. Discussion

To obtain synthetic polyploids, some chemical mutagens, especially colchicine, are most effective. Colchicine prevents spindle formation during mitosis, which prevents the separation of daughter chromosomes in anaphase and cytokinesis, leading to a doubling of the number of chromosomes in the cell [49]. In this study, to increase the number of chromosomes (autopolyploidy), representatives of the diploid cotton species *G. herbaceum* were treated with 0.1 and 0.2% solutions of colchicine and synthetic tetraploid plants were developed. In studies conducted with various percentage solutions of colchicine, it was found that a 0.2% solution of colchicine was more effective than a 0.1% solution. As an effective concentration, we recommend the use of a 0.2% (20 h) of a solution of colchicine to conduct this kind of research.

A different genetic variability was observed in F_2_C hybrid plants. Two transgressive plants with long fiber of 35.1–37.0 mm, and one transgressive plant with extra-long fiber of 39.1–41.0 mm were identified in the F_2_C progeny of *G. herbaceum* subsp. *frutescens* × *G. mustelinum* combination. The observed genetic variability, especially in cotton polyploid genotypes with long and extra-long fiber, has direct implications for cotton breeding. These variations would provide opportunities for selecting and developing cultivars with enhanced fiber qualities, potentially leading to the creation of commercially valuable cotton varieties with improved fiber length. According to the data mentioned above [43,44,45], although the F_2_C allopolyploid hybrid combination obtained with *G. mustelinum* (subsp. *frutescens* × *G. mustelinum*) is late maturing, fiber yield is low compared to other forms, it can be concluded that the release of long fiber and extra-long fiber plants is the effect of *G. mustelinum* genes. Another reason for this outcome can be explained by the mutation of some sections (gene alleles) in the genome of *G. herbaceum* due to the application of the experimental polyploidy method, and the dis-appearance or appearance of F_1_C allopolyploid hybrid genome regions. That is, depending on the distribution of the signs of parental forms in the hybrids of the second generation, it is estimated that the number of polymer genes and their constant state in future generations. These isolated forms will serve as a unique genetic resource for the development of long fiber varieties in future breeding programs. For this reason, we have planned to introduce specific F_2_C polyploids into conventional and molecular cotton breeding programs to obtain new varieties with desired traits through hybridization and backcrossing approaches. The parental genotypes possess distinct fiber properties, with *G. mustelinum* exhibiting soft and long fiber, while *G. herbaceum* has fiber with hygroscopic properties. The fiber characteristics mentioned above, valuable in the global cotton industry, are expected to be effectively incorporated into the future cultivars.

As a result of molecular and phylogenetic studies, it was shown that tetraploid hybrids derived from the *G. herbaceum* × *G. mustelinum* cross, are close to the species *G. mustelinum*. Also, it was found that compared with the first cluster of the phylogenetic tree, the second cluster appeared to be small. It only had one subcluster containing the forms *subsp. frutescens*, *subsp. pseudoarboreum f. harga*, and subsp. *africanum* belonging to the species *G. herbaceum* L., which indicates their phylogenetic relatedness. At the same time, it was noted that these forms are significantly distant from the type of *G. mustelinum*. Another important aspect of the research results was that *subsp. frutescens* from representatives of *G. herbaceum* are close to their cultivated forms, and that its subsp. *africanum* is a wilder form.

## 4. Materials and Methods

### 4.1. Plant Materials

In this study, wild, semi-wild and cultivated subspecies of diploid cotton *G. herbaceum* L. and wild tetraploid cotton *G. mustelinum* Miers ex Watt as well as their five interspecific hybrids (1) *G. herbaceum* subsp. *frutescens* × *G. mustelinum*; (2) *G. herbaceum* subsp. *pseudoarboreum* × *G. mustelinum*; (3) *G. herbaceum* subsp. *pseudoarboreum* f. *harga* × *G. mustelinum*; (4) *G. herbaceum* subsp. *africanum* × *G. mustelinum*; (5) *G. herbaceum* subsp. *euherbaceum* (variety A-833) × *G. mustelinum* were used. The seeds of parental genotypes were taken from the special cotton germplasm collection at the Institute of Genetics and Plant Experimental Biology (IGPEB) Academy of Sciences of the Republic of Uzbekistan (ASRUz). Experiments were conducted during 2020–2022 in the laboratory of Experimental polyploidy and phylogeny of cotton and the nursery at the IGPEB, located in Qibray district, Tashkent.

### 4.2. Hybridization and Polyploidization

Diploid (2n = 2x = 26) *G. herbaceum* L. subspecies were crossed with tetraploid (2n = 4x = 52) *G. mustelinum* Miers ex Watt and obtained five combinations of triploid (2n = 3x = 39) hybrid genotypes. Experiments were conducted under two conditions: (1) F_1_ hybrid seeds of triploids were treated with 0.1% colchicine for 24 h, and (2) 0.2% colchicine was applied for 20 h. Both conditions involved darkness at room temperature (22 °C) during seed germination, reaching approximately 1 cm in shoot length. Consequently, the duration of the colchicine treatment was reduced by four hours with an increased concentration. As a result, F_1_C (C for colchicine) synthetic allohexaploid (2n = 6x = 78) hybrid (*G. herbaceum* subsp. *frutescens* × *G. mustelinum*; *G. herbaceum* subsp. *pseudoarboreum* × *G. mustelinum*; *G. herbaceum* subsp. *pseudoarboreum* f. *harga* × *G. mustelinum*; *G. herbaceum* subsp. *africanum* × *G. mustelinum*; *G. herbaceum* subsp. *euherbaceum* (variety A-833) × *G. mustelinum*) genotypes have been obtained. Synthetic allohexaploid genotypes were planted aim to obtain F_2_C generations. Unfortunately, seed of two allohexaploid genotypes (*G. herbaceum* subsp. *pseudoarboreum* × *G. mustelinum* and *G. herbaceum* subsp. *euherbaceum* (variety A-833) × *G. mustelinum*) died at the germination stage. Two F_1_C allohexaploids (*G. herbaceum* subsp. *pseudoarboreum* f. *harga* × *G. mustelinum* and *G. herbaceum* subsp. *africanum* × *G. mustelinum*) were male-sterile. Only, F_1_C *G. herbaceum* subsp. *frutescens* × *G. mustelinum* allohexaploids were fertile and obtained F_2_C genotypes.

### 4.3. Cytological Analysis

Analysis of sporads and pollen viability were carried out according to Pausheva [50]. For the analysis of tetrads, 2–4 mm cotton buds (in the pinhead square and match-head square stages) were collected early morning and were fixed in the ethanol-acetic acid mixture (7:3 *v*/*v*). The samples were screened using a trinocular microscope (N-300M(MD101), Ningbo Yongxin Optics Co., Ltd., Ningbo, China) after staining with acetocarmine. For the analysis of tetrads, the meiotic index (Mi) was calculated as the percentage of normal tetrads over total sporads. In order to determine the pollen viability, pollen was collected from newly opened cotton flowers in the first half of the day and was stained with acetocarmine. Next cytological analysis was caried out using a Leica CM E microscope with a Leica EC3 camera (The Leica Microsystems Inc., Wetzlar, Germany).

### 4.4. Phenotypic Observation

During the 2021–2022 planting seasons, *G. herbaceum* L. subspecies, *G. mustelinum* Miers ex Watt and their F_1-2_C allohexaploids were evaluated for phenotypic traits in the field condition. To study morpho-biological traits, such as (i) vegetative growth period (VP); (ii) number of bolls per plant (NB); (iii) number of seeds per boll (NSB); (iv) boll weight (BW); (v) 1000-seed weight (SW); (vi) fiber length (FL); fiber strength (FS); and (vii) male fertility (MF) and sterility (MS) of pollens.

### 4.5. DNA Isolation and SSR Analysis

The genomic DNA from cotton plant leaf tissues was isolated using the cetyltrimethylammonium bromide (CTAB) method [51]. The DNA concentration was calculated by measuring the absorbance of 1 µL of the samples at 260/280 nm using the NanoDrop Eight spectrophotometer (Thermo Fisher Scientific, Waltham, MA, USA). DNA samples were diluted to a working concentration of 25 ng/µL. A total of 72 SSR (simple sequence repeat) markers were selected from CottonGen the cotton marker database (https://www.cottongen.org/data/markers) (accessed on 20 February 2022) [52]. PCR-based SSR genotyping was conducted as described previously [6,53,54]. The construction and visualization of the phylogenetic tree was performed using NCSS 12.

### 4.6. Statistical Analysis

Statistical analyses of the obtained experimental results were carried out according to Dospekhov [42]. The mean-square deviation from the mean was determined for the number of measurements *n* = 10 according to the formula:

$$\sigma =\surd \frac{\sum {\left(X_{i}-\bar{X}\right)}^{2}}{n-1}$$

Mean-square deviation from the mean value is calculated by the following formula:

$$\sigma_{\bar{X}}=\frac{\sigma }{\sqrt{n-1}}$$

Substituting the reliability criteria (Student’s coefficient) into the formula, the boundaries of the confidence interval for the arithmetic mean were obtained:

$$∆=t_{c}\sigma_{\bar{X}}$$

The relative error of the results of a series of *x* measurements at a confidence level of 95% will be equal to:

$$S=\frac{100\sigma_{\bar{X}}t_{c}}{\bar{X}}$$

*V*—the coefficient of variation was calculated by the below-mentioned formula:

(1) $$V=\frac{100\sigma_{\bar{X}}}{\bar{X}}$$

## 5. Conclusions

In this study, we made several crosses between cotton species and obtained interspecific triploid F_1_ hybrids in five different combinations: (1) *G. herbaceum* subsp. *frutescens* × *G. mustelinum*; (2) *G. herbaceum* subsp. *pseudoarboreum* × *G. mustelinum*; (3) *G. herbaceum* subsp. *pseudoarboreum* f. *harga* × *G. mustelinum*; (4) *G. herbaceum* subsp. *africanum* × *G. mustelinum*; (5) *G. herbaceum* subsp. *euherbaceum* (variety A-833) *× G. mustelinum*. Fertile allohexaploid F_1_C hybrids were obtained by polyploidization of triploid forms. Cytogenetic analysis showed the existence of univalent, open, and closed ring-shaped quadrivalent chromosomes at the stage of metaphase I in the F_1_C and F_2_C hybrids. Also, various meiotic anomalies (tetrad cells with micronuclei and polyads), as well as low rates of pollen (36.6–63.8%) and complete sterility (F_1_C subsp. *africanum* × G. *mustelinum*) were observed in the analysis of tetrads. This indicates the structural heterozygosity of polyploid hybrids.

A total of 57 out of the 72 SSR markers identified in the study were determined polymorphic between cotton species. Genomic changes were observed in allohexaploid hybrids. Allele changes were observed in F_1_C (*G. herbaceum* subsp. *frutescens* × *G. mustelinum*), F_1_C (*G. herbaceum* subsp. *pseudoarboreum* f. *harga* × *G. mustelinum*) and F_1_C (*G. herbaceum* subsp. *africanum* × *G. mustelinum*) hybrids, 31 (26.0%), 29 (24.3%) and 27 (22.6%) out of 119, 120 and 116 alleles, respectively. 

As a result of phylogenetic tree construction, the cotton accessions were clustered into two groups. The first group included three *Gossypium herbaceum* subspecies which are genetically similar to each other and subsp. *frutescens*. *G. mustelinum* and three interspecific allohexaploid hybrids F_1_C (*G. herbaceum* subsp. *frutescens* × *G. mustelinum*), F_1_C (*G. herbaceum* subsp. *pseudoarboreum* f. *harga* × *G. mustelinum*) and F_1_C (*G. herbaceum* subsp. *africanum* × *G. mustelinum*) were presented in the second group of the phylogenetic tree. Transgressive plants with long fiber were identified for potential improvement of fiber length in cultivated cottons. These synthetic allotetraploid cottons could also serve as valuable sources in the introgression of economically important traits including biotic and abiotic stress tolerance into the elite Upland cotton varieties.

## Figures and Tables

**Table 1:** The fertility and hybrid boll-setting rates in interspecific crosses between *G. herbaceum* and *G. mustelinum* species.

No.	Hybrid Combination	Number of Crosses	Number of Obtained Hybrid Bolls	Fertilization Rate, %	Boll Setting Rate, %			
					X ± Sx	Range	S	V
1.	*G. herbaceum* subsp. *africanum × G. mustelinum*	62	15	24.2	10.9 ± 1.0	7.7–18.2	3.06	28.09
2.	*G. herbaceum* subsp. *pseudoarboreum × G. mustelinum*	59	8	13.8	10.0 ± 1.9	4.3–23.1	5.9	58.7
3.	*G. herbaceum* subsp. *pseudoarboreum* f. *harga × G. mustelinum*	65	15	24.6	9.7 ± 1.1	5.9–15.4	3.39	34.95
4.	*G. herbaceum* subsp. *frutescens × G. mustelinum*	63	58	92.2	13.1 ± 3.2	4.0–41.7	10.12	77.25
5.	*G. herbaceum* subsp. *euherbaceum* (A-833) *× G. mustelinum*	62	33	54.5	16.3 ± 1.7	10.0–25.0	5.41	33.18

Note: S stands for the relative error of the results of a series of x measurements at a confidence level of 95%; and V stands for the coefficient of variation, as described by Dospekhov [42].

**Table 2:** The number of bolls per parental plant and the fertility of cotton seeds.

No.	Plant Samples	Number of Bolls		Number of Seeds Per Boll			Percentage of Complete Seeds Per Boll, %			
		Per Plant	Analyzed	Total	Complete	Empty	$\bar{x}$ ± S$\bar{x}$	Range	S	V %
Parental Lines										
1.	*G. herbaceum* subsp. *frutescens*	21	10	25.4	23.5	1.9	93 ± 2	84–100	6.17	6.7
2.	*G. herbaceum* subsp. *pseudoarboreum*	20	10	19.5	18.0	1.5	92 ± 2	85–100	6.00	6.5
3.	*G. herbaceum* subsp. *pseudoarboreum* f. *harga*	36	10	18.7	16.7	2.0	89 ± 2	80–100	5.90	6.6
4.	*G. herbaceum* subsp. *africanum*	27	10	18.5	17.5	1.0	95 ± 2	84–100	5.70	6.0
5.	*G. herbaceum* subsp. *euherbaceum* A-833	28	10	17.6	15.3	2.3	87 ± 2	82–100	6.08	7.0
6.	*G. mustelinum* Miers ex Watt	16	10	27.1	23.2	3.9	86 ± 2	77–96	6.62	8

Note: S stands for the relative error of the results of a series of x measurements at a confidence level of 95%; and V stands for the coefficient of variation, as described by Dospekhov [42].

**Table 3:** The number of bolls per hybrid plant of F_1_C and F_2_C polyploids.

No.	Hybrid Combination	Number of Bolls		Number of Seeds Per Boll			Percentage of Complete Seeds Per Boll, %			
		Per Plant	Analyzed	Total	Complete	Empty	$\bar{x}$ ± S$\bar{x}$	Range	S	V %
F_1_C polyploid hybrids										
1.	*G. herbaceum* subsp. *frutescens* × *G. mustelinum*	21	10	14.3	2.8	11.5	19 ± 2	8–27	6.13	31.6
2.	*G. herbaceum* subsp. *pseudoarboreum* f. *harga* × *G. mustelinum*	1	1	9	2	7	-	-	-	-
3.	*G. herbaceum* subsp. *africanum* × *G. mustelinum*	-	-	-	-	-	-	-	-	-
F_2_C polyploid hybrids										
4.	T 51-13 *G. herbaceum* subsp. *frutescens* × *G. mustelinum*	26	10	22.9	7.0	15.9	30 ± 1	25–36	3.75	12.3
5.	T 30-8 *G. herbaceum* subsp. *frutescens* × *G. mustelinum*	24	10	27.2	11.6	15.6	43 ± 1	38–46	2.50	5.9
6.	T 38-3 *G. herbaceum* subsp. *frutescens* × *G. mustelinum*	21	10	26.0	9.5	16.5	36 ± 1	31–43	3.69	10.3
7.	T 30-6 *G. herbaceum* subsp. *frutescens* × *G. mustelinum*	19	10	18.7	9.2	9.5	49 ± 2	41–61	6.60	13.4
8.	T 38-12 *G. herbaceum* subsp. *frutescens* × *G. mustelinum*	18	10	22.8	7.8	15.0	34 ± 1	28–39	3.78	11.1

Note: S stands for the relative error of the results of a series of x measurements at a confidence level of 95%; and V stands for the coefficient of variation, as described by Dospekhov [42].

**Table 4:** Heredity and variability of the vegetative growth duration in F_1_C and F_2_C—polyploid hybrids and parental lines.

No.	Plant Samples		Vegetative Growth Duration, Day				
			Range	$\bar{x}$ ± S$\bar{x}$	S	V %	hp
Parental lines							
1.	*G. herbaceum* subsp. *frutescens*		116–119	117.2 ± 0.4	1.3	1.1	-
2.	*G. herbaceum* subsp. *pseudoarboreum*		118–122	119.6 ± 0.4	1.5	1.3	-
3.	*G. herbaceum* subsp. *pseudoarboreum* f. *harga*		126–129	127.2 ± 0.4	1.3	1.0	-
4.	*G. herbaceum* subsp. *africanum*		135–139	137.4 ± 0.4	1.8	1.3	-
5.	*G. herbaceum* subsp. *euherbaceum* A-833		116–119	117.4 ± 0.4	1.3	1.1	-
6.	*G. mustelinum*		150–153	151.8 ± 0.4	1.3	0.9	-
F_1_C polyploid hybrids							
7.	*G. herbaceum* subsp. *frutescens* × *G. mustelinum*		125–133	128.6 ± 0.9	2.8	2.3	0.34
8.	*G. herbaceum* subsp. *pseudoarboreum* f. *harga* × *G. mustelinum*		137–142	139.6 ± 0.7	2.1	1.5	−0.01
9.	*G. herbaceum* subsp. *africanum* × *G. mustelinum*		142–150	145.2 ± 1.3	3.1	2.2	−0.08
F_2_C polyploid hybrids							
10.	*G. herbaceum* subsp. *frutescens* × *G. mustelinum*	81 plants n = 4	115–119	116.6 ± 0.5	1.8	1.6	-
			120–124	121.8 ± 0.7	2.1	1.7	-
			125–129	127.8 ± 0.5	1.6	1.3	-
			130–134	131.4 ± 0.6	2.0	1.5	-
			135–139	136.8 ± 0.7	2.1	1.5	-
			140–144	141.6 ± 0.5	1.5	1.1	-
			145–149	147.2 ± 0.5	1.6	1.1	-
			150–155	152.2 ± 0.8	2.6	1.7	-

Note: S stands for the relative error of the results of a series of x measurements at a confidence level of 95%; and V stands for the coefficient of variation, as described by Dospekhov [42].

**Table 5:** Fiber length and strength in F_1_C and F_2_C—polyploid hybrids and parental lines.

No.	Plant Samples	Fiber Length, mm					Fiber Strength, cN/tex
		Range	$\bar{x}$ ± S$\bar{x}$	S	V %	hp	
Parental lines							
1.	*G. herbaceum* subsp. *frutescens*	16.0–18.0	17.2 ± 0.2	0.63	3.7	-	35.5
2.	*G. herbaceum* subsp. *pseudoarboreum*	24.0–27.0	25.1 ± 0.4	1.20	4.8	-	
3.	*G. herbaceum* subsp. *pseudoarboreum* f. *harga*	19.0–21.0	20.1 ± 0.2	0.74	3.7	-	21.4
4.	*G. herbaceum* subsp. *africanum*	24.0–26.0	24.7 ± 0.3	0.82	3.3	-	32.6
5.	*G. herbaceum* subsp. *euherbaceum* A-833	24.0–26.0	25.2 ± 0.3	0.79	3.1	-	27.2
6.	*G. mustelinum*	25.0–27.0	25.6 ± 0.2	0.70	2.7	-	17.3
F_1_C polyploid hybrids							
7.	*G. herbaceum* subsp. *frutescens* × *G. mustelinum*	25.0–32.0	28.7 ± 0.9	0.91	3.1	1.74	-
F_2_C polyploid hybrids							
8.	*G. herbaceum* subsp. *frutescens* × *G. mustelinum*	23.1–41.0	29.7 ± 1.2	3.3	11.0	57.3	-

Note: S stands for the relative error of the results of a series of x measurements at a confidence level of 95%; and V stands for the coefficient of variation, as described by Dospekhov [42].

**Table 6:** Conjugation of cotton chromosomes at metaphase-I stage of meiosis.

No.	Hybrid Combinations	The Number of Studied Maternal Anther Cells	Average Number Per Cell		
			Univalents	Bivalents	Quadrivalents
	F_1_C polyploid hybrids				
1.	*G. herbaceum* subsp. *frutescens* × *G. mustelinum*	22	0.45 ± 0.29	38.22 ± 0.25	0.36 ± 0.14
2.	*G. herbaceum* subsp. *pseudoarboreum* f. *harga* × *G. mustelinum*	15	1.73 ± 0.85	37.53 ± 0.44	0.46 ± 0.24
3.	*G. herbaceum* subsp. *africanum* × *G. mustelinum*	16	1.81 ± 0.84	37.56 ± 0.43	0.50 ± 0.24
	F_2_C polyploid hybrids				
4.	T 37-14 *G. herbaceum* subsp. *frutescens* × *G. mustelinum*	30	1.46 ± 0.55	24.73 ± 0.29	0.26 ± 0.09
5.	T 38-3 *G. herbaceum* subsp. *frutescens* × *G. mustelinum*	23	-	26.00 ± 0.00	-
6.	T 37-4 *G. herbaceum* subsp. *frutescens* × *G. mustelinum*	28	0.64 ± 0.25	38.18 ± 0.23	0.25 ± 0.11
7.	T 49-2 *G. herbaceum* subsp. *frutescens* × *G. mustelinum*	22	0.54 ± 0.2 7	38.00 ± 0.2 7	0.27 ± 0.13
8.	T 30-8 *G. herbaceum* subsp. *frutescens* × *G. mustelinum*	18	-	26.00 ± 0.00	-

**Table 7:** Cytological observation of F_1_C generation polyploid hybrids.

No.	Hybrid Combinations	Total Number of Spores	Meiotic Index, %	Micronuclear Tetrads, %	Polyads, %
	F_1_C polyploid hybrids				
1.	*G. herbaceum* subsp. *frutescens* × *G. mustelinum*	612	90.36 ± 1.19	3.27 ± 0.72	6.37 ± 0.98
2.	*G. herbaceum* subsp. *pseudoarboreum* f. *harga* × *G. mustelinum*	219	96.80 ± 1.18	-	3.19 ± 1.18
3.	*G. herbaceum* subsp. *africanum* × *G. mustelinum*	481	96.05 ± 0.88	0.83 ± 0.41	3.12 ± 0.79
	F_2_C polyploid hybrids				
4.	T 1-8 *G. herbaceum* subsp. *frutescens* × *G. mustelinum*	411	96.15 ± 0.12	0.89 ± 0.41	3.07 ± 0.72
5.	T 38-12 *G. herbaceum* subsp. *frutescens* × *G. mustelinum*	325	97.05 ± 0.64	-	2.62 ± 0.29
6.	T 30-6 *G. herbaceum* subsp. *frutescens* × *G. mustelinum*	354	97.21 ± 0.47	-	2.78 ± 0.71
7.	T 51-13 *G. herbaceum* subsp. *frutescens* × *G. mustelinum*	298	97.45 ± 0.88	0.69 ± 0.23	2.11 ± 0.41
8.	T 30-7 *G. herbaceum* subsp. *frutescens* × *G. mustelinum*	341	98.51 ± 0.48	-	1.48 ± 0.44
9.	T 30-8 *G. herbaceum* subsp. *frutescens* × *G. mustelinum*	405	96.05 ± 0.88	-	3.12 ± 0.79
10.	T 38-3 *G. herbaceum* subsp. *frutescens* × *G. mustelinum*	357	98.54 ± 0.81	-	1.42 ± 0.38

**Table 8:** Development of cotton pollens in F_1_C polyploid hybrids.

No.	Hybrid Combinations	Total Number of Pollens	Pollen Fertility, %
1.	*G. herbaceum* subsp. *frutescens* × *G. mustelinum*	324	32.7 ± 3.6
2.	*G. herbaceum* subsp. *pseudoarboreum* f. *harga* × *G. mustelinum*	432	63.9 ± 3.6
3.	*G. herbaceum* subsp. *africanum* × *G. mustelinum*	541	0

**Table 9:** Analysis of pollen quality in amphidiploid hybrids of F_2_C *G. herbaceum* subsp. *frutescens × G. mustelinum*.

No.	Sample	Total Number of Pollens	Pollen Fertility, %	No.	Sample	Total Number of Pollens	Pollen Fertility, %
1.	T 1-13	1199	33.9 ± 1.9	13.	T 30-8	2955	55.7 ± 0.8
2.	T 1-4	700	40.4 ± 3.4	14.	T 37-4	482	50.2 ± 5.2
3.	T 1-8	482	51.0 ± 5.2	15.	T 37-8	796	23.1 ± 2.2
4.	T 30-23	538	27.1 ± 3.7	16.	T 38-12	846	60.9 ± 2.8
5.	T 30-6	666	60.2 ± 3.6	17.	T 38-3	1315	67.8 ± 1.7
6.	T 30-7	738	65.9 ± 3.0	18.	T 38-4	367	38.4 ± 6.4
7.	T 37-14	114	7.9 ± 6.4	19.	T 38-7	352	10.2± 2.6
8.	T 30-1	267	22.9 ± 6.6	20.	T 42-2	159	14.5 ± 7.8
9.	T 33-2	690	46.8 ± 3.6	21.	T 49-2	606	1.5 ± 0.2
10.	T 33-6	248	5.6 ± 21.1	22.	T 51-15	426	34.3 ± 5.3
11.	T 36-1	76	15.8 ± 17.5	23.	T 51-19	363	25.1 ± 5.2
12.	T 37-2	228	17.5 ± 6.3	24.	T 51-13	1536	51.8 ± 1.6

## Data Availability

Data is contained within the article and Supplementary Materials.

## Supplementary Materials

The following supporting information can be downloaded at: https://www.mdpi.com/article/10.3390/plants12244184/s1, Table S1: The panel of SSR markers associated with economically important traits; Table S2: Polymorphic SSR markers, and their polymorphism information content (PIC) and heterozygosity (He) values. References [55,56,57,58,59,60,61,62,63,64,65,66,67,68,69,70,71,72,73,74,75,76,77,78,79,80,81,82,83,84,85,86,87,88,89,90,91,92,93,94,95,96,97,98] are cited in the supplementary materials.

## References

[1] Fryxell P. (1979). The Natural History of the Cotton Tribe (Malvaceae, Tribe Gossypieae).

[2] Wendel J.F., Grover C.E., Fang D.D., Percy R.G. (2015). Taxonomy and evolution of the cotton genus, *Gossypium*. Cotton.

[3] Wendel J., Brubaker C., Alvarez I., Cronn R., Stewart J., Paterson A.H. (2009). Evolution and natural history of the cotton genus. Genetics and Genomics of Cotton.

[4] Brubaker C., Paterson A., Wendel J. (1999). Comparative genetic mapping of allotetraploid cotton and its diploid progenitors. Genome.

[5] Wendel J., Cronn R. (2003). Polyploidy and the evolutionary history of cotton. Adv. Agron..

[6] Kushanov F., Komilov D., Turaev O., Ernazarova D., Amanboyeva R., Gapparov B., Yu J. (2022). Genetic analysis of mutagenesis that induces the photoperiod insensitivity of wild cotton *Gossypium hirsutum* subsp. purpurascens. Plants.

[7] Kushanov F., Turaev O., Ernazarova D., Gapparov B., Oripova B., Kudratova M., Rafieva F., Khalikov K., Erjigitov D., Khidirov M. (2021). Genetic diversity, QTL mapping, and marker-assisted selection technology in cotton (*Gossypium* spp.). Front. Plant Sci..

[8] Altman D. (1988). Exogenous hormone applications at pollination for in vitro and in vivo production of cotton interspecific hybrids. Plant Cell Rep..

[9] Meyer V. (1974). Interspecific cotton breeding. Econ. Bot..

[10] Montes E., Coriton O., Eber F., Huteau V., Lacape J., Reinhardt C., Marais D., Hofs J., Chevre A., Pannetier C. (2017). Assessment of gene flow between *Gossypium hirsutum* and *G. herbaceum*: Evidence of unreduced gametes in the diploid progenitor. G3.

[11] Huang G., Wu Z., Percy R.G., Bai M., Li Y., Frelichowski J.E., Hu J., Wang K., Yu J.Z., Zhu Y. (2020). Genome sequence of *Gossypium herbaceum* and genome updates of *Gossypium arboreum* and *Gossypium hirsutum* provide insights into cotton A-genome evolution. Nat. Genet..

[12] Pershina L. (2009). On the role of distant hybridization and polyploidy in plant evolution. Vogis Bull..

[13] Alam H., Razaq M. (2015). Induced polyploidy as a tool for increasing tea (*Camellia sinensis* L.) production. Northeast Agric. Univ..

[14] Vavilov N. (1965). Doubling the number of chromosomes as a method for obtaining new plant forms. Sel. Work..

[15] Kamburova V., Salakhutdinov I., Shermatov S., Buriev Z., Abdurakhmonov I. (2021). Cotton as a Model for Polyploidy and Fiber Development Study. Model Organisms in Plant Genetics.

[16] Bae S., Islam M., Kim H., Lim K. (2020). Induction of tetraploidy in watermelon with oryzalin treatments. Hort. Sci. Technol..

[17] Dasgeb B., Kornreich D., McGuinn K., Okon L., Brownell I., Sackett D. (2018). Colchicine: An ancient drug with novel applications. Br. J. Derm..

[18] Stanys V., Staniene G., Siksnianas T. (2004). In vitro induction of ploidy in Ribes. Acta Uni. Latv. Biol..

[19] Petersen K., Hagberg P., Kristiansen K. (2003). Colchicine and oryzalin mediated chromosome doubling in different genotypes of Miscanthus sinensis. Plant Cell Tis. Organ. Cult..

[20] Vainola A. (2000). Polyploidization and early screening of Rhododendron hybrids. Euphytica.

[21] Jia Y.H., Sun J.L., Wang X.W., Zhou Z.L., Pan Z.E., He S.P., Pang B.Y., Wang L.R. (2013). Molecular diversity and association analysis of drought and salt tolerance in *G.hirsutum* L. germplasm. J. Integ. Agr..

[22] Abdurakhmonov I.Y. (2014). World Cotton Germplasm Resources.

[23] Campbell B., Saha S., Percy R., Frelichowski J., Jenkins J., Park W., Mayee C., Gotmare V., Dessauw D., Giband M. (2010). Status of the global cotton germplasm resources. Crop Sci..

[24] Abdurakhmonov I., Kohel R., Yu J., Pepper A., Abdullaev A., Kushanov F., Salakhutdinov I., Buriev Z., Saha S., Scheffler B. (2008). Molecular diversity and association mapping of fiber quality traits in exotic *G.hirsutum* L. germoplasm. Genomics.

[25] Chen Q., Wang W., Wang C., Zhang M., Yu J., Zhang Y., Yuan B., Ding Y., Jones D.C., Paterson A. (2020). Validation of QTLs for fiber quality introgressed from *Gossypium mustelinum* by selective genotyping. G3.

[26] Jareczek J., Grover C., Hu G., Xiong X., Arick I., Peterson D., Wendel J. (2023). Domestication over speciation in allopolyploid cotton species: A stronger transcriptomic pull. Genes.

[27] Grover C.E., Yoo M.J., Lin M., Murphy M.D., Harker D.B., Byers R.L., Lipka A.E., Hu G., Yuan D., Conover J.L. (2020). Genetic analysis of the transition from wild to domesticated cotton (*Gossypium hirsutum* L.). G3.

[28] Jena S., Srivastava A., Rai K., Ranjan A., Singh S., Nisar T. (2012). Development and characterization of genomic and expressed SSRs for levant cotton (*Gossypium herbaceum* L.). Theor. App. Genet..

[29] Barroso P., Hoffmann L., Batista C., Freitas R., Alves M., Silva U., Andrade F. (2010). In situ conservation and genetic diversity of three populations of *Gossypium mustelinum* Miers (exWatt). Genet. Res. Crop Evol..

[30] Wang B., Zhuang Z., Zhang Z., Draye X., Shuang L.-S., Shehzad T., Lubbers E.L., Jones D., May O.L., Paterson A.H. (2017). Advanced backcross QTL analysis of fiber strength and fineness in a cross between *Gossypium hirsutum* and *G. mustelinum*. Front. Plant Sci..

[31] Yang Y., You C., Wang N., Wu M., Le Y., Wang M., Zhang X., Yu Y., Lin Z. (2023). *Gossypium mustelinum* genome and an introgression population enrich interspecific genetics and breeding in cotton. Theor. Appl. Genet..

[32] Sun Y., Zhang X., Nie Y., Guo X., Jin S., Liang S. (2004). Production and characterization of somatic hybrids between Upland cotton (*Gossypium hirsutum*) and wild cotton (*G. klotzschianum* Anderss) via electrofusion. Theor. App. Genet..

[33] Yu J.Z., Kohel R.J., Fang D.D., Cho J., Van A.D., Ulloa M., Hoffman S.M., Pepper A.E., Stelly D.M., Jenkins J.N. (2012). A high-density simple sequence repeat and single nucleotide polymorphism genetic map of the tetraploid cotton genome. G3.

[34] Newaskar G., Chimote V., Mehetre S., Jadhav S. (2013). Interspecific hybridization in *Gossypium* L.: Characterization of progenies with different ploidy-confirmed multigenomic backgrounds. Plant Breed..

[35] Zhang X., Zhai C., He L., Guo Q., Zhang X., Xu P., Su H., Gong Y., Ni W., Xinlian S. (2014). Morphological, cytological and molecular analyses of a synthetic hexaploid derived from an interspecific hybrid between *Gossypium hirsutum* and *Gossypium anomalum*. Crop J..

[36] Liu Q., Chen Y., Chen Y., Wang Y., Chen J., Zhang T., Zhang T., Zhouet B. (2015). A new synthetic allotetraploid (A_1_A_1_G_2_G_+_) between *Gossypium herbaceum* and *G. australe*: Bridging for simultaneously transferring favorable genes from these two diploid species into Upland cotton. PLoS ONE.

[37] Chen Y., Wang Y., Wang K., Zhu X., Guo W., Zhang T., Zhou B. (2014). Construction of a complete set of alien chromosome addition lines from *Gossypium australe* in *Gossypium hirsutum*: Morphological, cytological, and genotypic characterization. Theor. App. Genet..

[38] Chen Y., Wang Y., Zhao T., Yang J., Feng S., Nazeer W. (2015). A new synthetic amphiploid (AADDAA) between *G. hirsutum* and *G. arboreum* lays the foundation for transferring resistances to verticillium and drought. PLoS ONE.

[39] Chen D., Wu Y., Zhang X., Li F. (2015). Analysis of [*Gossypium capitis-viridis* × (*G.hirsutum × G.australe*)^2^] trispecific hybrid and selected characteristics. PLoS ONE.

[40] Kushanov F., Buriev Z., Shermatov S., Turaev O., Norov T., Pepper A., Saha S., Ulloa M., Yu J., Jenkins J. (2017). QTL mapping for flowering-time and photoperiod insensitivity of cotton *Gossypium darwinii* Watt. PLoS ONE.

[41] Yin X., Zhan R., He Y., Song S., Wang L., Ge Y., Chen D. (2020). Morphological description of a novel synthetic allotetraploid(A_1_A_1_G_3_G_3_) of *Gossypium herbaceum* L. and *G. nelsonii* Fryx. suitable for disease-resistant breeding applications. PLoS ONE.

[42] Dospekhov B.A. (1985). Methodology of Field Experience (with the Basics of Statistical Processing of Research Results).

[43] Wang B., Draye X., Zhuang Z., Zhang Z., Liu M., Lubbers E.L., Jones D., May O.L., Paterson A.H., Chee P.W. (2017). QTL analysis of cotton fiber length in advanced backcross populations derived from a cross between *Gossypium hirsutum* and *G. mustelinum*. Theor. Appl. Genet..

[44] Wang B., Liu L., Zhang D., Zhuang Z., Guo H., Qiao X., Wei L., Rong J., May O.L., Paterson A.H. (2016). A genetic map between *Gossypium hirsutum* and the Brazilian endemic *G. mustelinum* and its application to QTL mapping. G3.

[45] Wang B., Draye X., Zhang Z., Zhuang Z., May O.L., Paterson A.H., Chee P.W. (2016). Advanced backcross quantitative trait locus analysis of fiber elongation in a cross between *Gossypium hirsutum* and *G. mustelinum*. Crop Sci..

[46] Shkutina F.M., Khvostova V.V., Bogdanov Y.F. (1975). Meiosis in distant hybrids and amphidiploids. V. Sat. Cytology and Genetics of Meiosis.

[47] Kozak M.F. (2004). Mitotic rhythms in representatives of the genus *Glycine* L. Tsitol Genet..

[48] Sanamyan M.F. (1988). Cytogenetic study of hybrids and mutants of cotton. Abstract Candidate of Biological Sciesnces.

[49] Blasio F., Prieto P., Pradillo M., Naranjo T. (2022). Genomic and meiotic changes accompanying polyploidization. Plants.

[50] Pausheva Z.P. (1988). Workshop on Cytology.

[51] Paterson A.H., Brubaker C.L., Wendel J.F. (1993). A rapid method for extraction of cotton (*Gossypium* spp.) genomic DNA suitable for RFLP or PCR analysis. Plant Mol. Biol. Rep..

[52] Yu J., Jung S., Cheng C., Lee T., Zheng P., Buble K., Crabb J., Humann J., Hough H., Jones D. (2021). CottonGen: The community database for cotton genomics, genetics, and breeding research. Plants.

[53] Adylova A., Norbekov G., Khurshut E., Nikitina E., Kushanov F. (2018). SSR analysis of the genomic DNA of perspective Uzbek hexaploid winter wheat varieties. Vavilov J. Genet. Breed..

[54] Turaev O.S., Baboev S.K., Ziyaev Z.M., Norbekov J.K., Erjigitov D.S., Bakhadirov U.S., Tursunmurodova B.T., Dolimov A.A., Turakulov K.S., Ernazarova D.K. (2023). Present status and future perspectives of wheat (*Triticum aestivum* L.) research in Uzbekistan. SABRAO J. Breed. Genet..

[55] Shen X., Guo W., Zhu X., Yu J., Kohel R., Zhang T. (2005). Molecular mapping of QTLs for fiber qualities in three diverse lines in Upland cotton using SSR markers. Mol. Breed..

[56] Wu J., Gutierrez O., Jenkins J., McCarty J., Zhu J. (2008). Quantitative analysis and QTL mapping for agronomic and fiber traits in an RIL population of Upland cotton. Euphytica.

[57] Sun F.D., Zhang J.H., Wang S.F., Gong W.K., Shi Y.Z., Liu A.Y., Yuan Y.L. (2011). QTL mapping for fiber quality traits across multiple generations and environments in Upland cotton. Mol. Breed..

[58] Liu H., Quampah A., Chen J., Li J., Huang Z., He Q., Zhu S. (2017). QTL mapping with different genetic systems for nine non-essential amino acids of cottonseeds. Mol. Genet. Genom..

[59] Ma L., Zhao Y., Wang Y., Shang L., Hua J. (2017). QTLS analysis and validation for fiber quality traits using maternal backcross population in Upland cotton. Fron. Plant Sci..

[60] An C., Jenkins J.N., Wu J., Guo Y., McCarty J.C. (2009). Use of fiber and fuzz mutants to detect QTL for yield components, seed, and fiber traits of Upland cotton. Euphytica.

[61] Du L., Cai C., Wu S., Zhang F., Hou S., Guo W. (2016). Evaluation and exploration of favorable QTL alleles for salt stress related traits in cotton cultivars (*G. hirsutum* L.). PLoS ONE.

[62] Abdelraheem A., Zhu Y., Zhang J. (2022). Quantitative trait locus mapping for fusarium wilt race 4 resistance in a recombinant inbred line population of pima cotton (*Gossypium barbadense*). Pathogens.

[63] Liang Q., Li P., Hu C., Hua H., Li Z., Rong Y., Wang K., Hua J. (2014). Dynamic QTL and epistasis analysis on seedling root traits in Upland cotton. J. Genet..

[64] Liang Q., Hu C., Hua H., Li Z., Hua J. (2013). Construction of a linkage map and QTL mapping for fiber quality traits in Upland cotton (*Gossypium hirsutum* L.). Chinese Sci. Bull..

[65] Shao Q., Zhang F., Tang S., Liu Y., Fang X., Liu D., Zhang Z. (2014). Identifying QTL for fiber quality traits with three Upland cotton (*Gossypium hirsutum* L.) populations. Euphytica.

[66] Shang L., Liu F., Wang Y., Abduweli A., Cai S., Wang K., Hua J. (2015). Dynamic QTL mapping for plant height in Upland cotton (*Gossypium hirsutum*). Plant Breed..

[67] Wang F., Zhang C., Liu G., Chen Y., Zhang J., Qiao Q., Zhang J. (2016). Phenotypic variation analysis and QTL mapping for cotton (*Gossypium hirsutum* L.) fiber quality grown in different cotton-producing regions. Euphytica.

[68] Wang B., Guo W., Zhu X., Wu Y., Huang N., Zhang T. (2007). QTL mapping of yiyeld and yiyeld components for elite hybrid derived-RILs in Upland cotton. Genet. Genom..

[69] Shen X., Guo W., Lu Q., Zhu X., Yuan Y., Zhang T. (2007). Genetic mapping of quantitative trait loci for fiber quality and yield trait by RIL approach in Upland cotton. Euphytica.

[70] Ulloa M., Wang C., Hutmacher R., Wright S., Davis R., Saski C., Roberts P. (2011). Mapping Fusarium wilt race 1 resistance genes in cotton by inheritance, QTL and sequencing composition. Mol. Genet. Genet..

[71] Jamshed M., Jia F., Gong J., Palanga K., Shi Y., Li J., Shang H., Liu A., Chen T., Zhang Z. (2016). Identification of stable quantitative trait loci (QTLs) for fiber quality traits across multiple environments in *Gossypium hirsutum* recombinant inbred line population. BMC Genomics.

[72] Wang B., Wu Y., Huang N., Zhu X., Guo W., Zhang T. (2006). QTL Mapping for Plant Architecture Traits in Upland Cotton Using RILs and SSR Markers. Actu Genet. Sinica.

[73] Abdelraheem A., Kuraparthy V., Zhang J. Identification of drought and salt tolerant cotton germplasm and associated markers in the U.S. Upland germplasm. Proceedings of the ASA, CSSA and SSSA International Annual Meetings.

[74] Deng X., Gong J., Liu A., Shi Y., Gong W., Ge Q., Li J., Shang H., Wu Y., Yuan Y. (2019). QTL mapping for fiber quality and yield-related traits across multiple generations in segregating population of CCRI 70. J. Cotton Res..

[75] Li H., Pan Z., He S., Jia Y., Geng X., Chen B., Wang L., Pang B., Du X. (2021). QTL mapping of agronomic and economic traits for four F_2_ populations of Upland cotton. J. Cotton Res..

[76] Qin Y.-S., Liu R.-Z., Mei H.-X., Zhang T.-Z., Guo W.-Z. (2009). QTL Mapping for yield traits in Upland cotton (*Gossypium hirsutum* L.). Acta Agron. Sin..

[77] Yu J., Ulloa M., Hoffman S., Kohel R., Pepper A., Fang D., Percy R., Burke J. (2014). Mapping genomic loci for cotton plant architecture, yield components, and fiber properties in an interspecific (*Gossypium hirsutum* L. × *G. barbadense* L.) RIL population. Mol. Genet. Genom..

[78] Shang L., Liang Q., Wang Y., Wang X., Wang K., Abduweli A., Ma L., Cai S., Hua J. (2015). Identification of stable QTLs controlling fiber traits properties in multi-environment using recombinant inbred lines in Upland cotton (*Gossypium hirsutum* L.). Euphytica.

[79] Yu X., Chu B., Liu R., Sun J., Brian J., Wang H., Shuijin Z., Sun Y. (2012). Characteristics of fertile somatic hybrids of *G. hirsutum* L. and *G. trilobum* generated via protoplast fusion. Theor. Appl. Genet..

[80] Yu Y., Yuan D., Liang S., Li X., Wang X., Lin Z., Zhang X. (2011). Genome structure of cotton revealed by a genome-wide SSR genetic map constructed from a BC1 population between *Gossypium hirsutum* and *G. barbadense*. BMC Genom..

[81] Zhang K., Zhang J., Ma J., Tang Y., Liu D., Teng Z. (2012). Genetic mapping and quantitative trait locus analysis of fiber quality traits using a three-parent composite population in Upland cotton (*Gossypium hirsutum* L.). Mol. Breeding.

[82] Zhang S., Feng L., Xing L., Yang B., Gao X., Zhu X., Zhang T., Zhou B., Jenkins J. (2016). New QTLs for lint percentage and boll weight mined in introgression lines from two feral landraces into *Gossypium hirsutum* acc TM-1. Plant Breed..

[83] Tan Z., Fang X., Tang S., Zhang J., Liu D., Teng Z., Li L., Ni H., Zheng F., Liu D. (2015). Genetic map and QTL controlling fiber quality traits in Upland cotton (*Gossypium hirsutum* L.). Euphytica.

[84] Yu W., Zhang K., Li S., Yu H., Zhai M., Wu M., Li X., Fan S., Song M., Yang D. (2013). Mapping quantitative trait loci for lint yield and fiber quality across environments in a *Gossypium hirsutum × Gossypium barbadense* backcross inbred line population. Theor. Appl. Genet..

[85] Yu J., Kohel R., Smith C. (2010). The construction of a tetraploid cotton genome wide comprehensive reference map. Genomics.

[86] Qin H., Guo W., Zhang M., Zhang T. (2008). QTL mapping of yield and fiber traits based on a four-way cross population in *Gossypium hirsutum* L. Theor. App. Genet..

[87] Xiao J., Wu K., Fang D., Stelly D., Yu J., Cantrell R. (2009). New SSR markers for use in cotton (*Gossypium* spp.). Imp. J. Cotton Sci..

[88] Shi Y., Zhang B., Liu A., Li W., Li J., Lu Q., Zhang Z., Li S., Gong W., Shang H. (2016). Quantitative trait loci analysis of Verticillium wilt resistance in interspecific backcross populations of *Gossypium hirsutum × Gossypium barbadense*. BMC Genomics.

[89] Wang C., Ulloa M., Duong T., Roberts P.A. (2018). Quantitative trait loci mapping of multiple independent loci for resistance to *fusarium oxysporum* f.sp. vasinfectum races 1 and 4 in an interspecific cotton population. Phytopathology.

[90] Li S., Liu A., Kong L., Gong J., Li J., Gong W., Lu Q., Li P., Shang H., Xiao X. (2019). QTL mapping and genetic effect of chromosome segment substitution lines with excellent fiber quality from *Gossypium hirsutum × Gossypium barbadense*. Mol. Genet. Genom..

[91] Wang Y., Ning Z., Hu Y., Chen J., Zhao R., Chen H., Ai N., Guo W., Zhang T. (2015). Molecular mapping of restriction-site associated DNA markers in allotetraploid Upland cotton. PLoS ONE.

[92] Nie X., Huang C., You C., Li W., Zhao W., Shen C., Zhang B., Wang H., Yan Z., Dai B. (2016). Genome-wide SSR-based association mapping for fiber quality in nation-wide Upland cotton inbreed cultivars in China. BMC Genomics.

[93] Muhammad S., Anam T., Shagufta P., Amina A., Sumera A., Muhammad R., Xianliang S., Xuezhen S., Tianzhen Z. (2019). Population structure, linkage disequilibrium and association mapping for cotton leaf curl disease incidence in cotton (*Gossypium hirsutum* L.). Research Square.

[94] Gutierrez O.A., Robinson A.F., Jenkins J.N., McCarty J.C., Wubben M.J., Callahan F.E., Nichols R.L. (2010). Identification of QTL regions and SSR markers associated with resistance to reniform nematode in *Gossypium barbadense* L. accession GB713. Theor. App. Genet..

[95] Huang C., Shen C., Wen T., Gao B., Zhu D., Li X., Ahmed M., Li D., Lin Z. (2018). SSR-based association mapping of fiber quality in Upland cotton using an eight-way MAGIC population. Mol. Genet. Genom..

[96] Ma L., Su Y., Wang Y., Nie H., Cui Y., Cheng C., Wang M., Hua J. (2019). QTL and genetic analysis controlling fiber quality traits using paternal backcross population in Upland Cotton. J. Cotton Res..

[97] Zhao L., Lv D., Cai C., Tong C., Chen D., Zhang W., Du H., Guo H., Guo Z. (2012). Toward allotetraploid cotton genome assembly: Integration of a high-density molecular genetic linkage map with DNA sequence information. BMC Genomics.

[98] Han Z., Wang C., Song X., Guo W., Gou J., Li C., Chen X., Zhang T. (2006). Characteristics, development and mapping of *Gossypium hirsutum* derived EST-SSRs in allotetraploid cotton. Theor. App. Genet..

