# Supplementary material for: Genomic and Cytogenetic Analysis of Synthetic Polyploids between Diploid and Tetraploid Cotton (Gossypium) Species
Source: Plants (Basel). 2023 Dec 17;12(24):4184. doi: 10.3390/plants12244184 (PMC10748080; doi:10.3390/plants12244184)
Supplement: Supplementary file 1 [file plants-12-04184-s001.zip › plants-2718873-supplementary.pdf]

Table S1. The panel of SSR markers associated with economically important traits

No.	SSR marker	Chr. No.*	Reference
1	BNL1122	D16, A07	[56]
2	BNL1421	A13	[57]
3	BNL1694	D16, A07	[57,58]
4	BNL2496	D17	[59]
5	BNL3140	D23, A09	[56]
6	BNL3171	D21	[60]
7	BNL3347	A05	[61]
8	BNL3436	D25	[56]
9	BNL3452	D19, A05	[62]
10	BNL3594	A06	[62]
11	BNL3601	A05	[57]
12	BNL3792	A08	[62]
13	BNL3932	A06, A02	[63]
14	BNL4030	D22	[64]
15	CGR5602	A11	[65]
16	CGR5732	D19, A05	[66]
17	CGR5758	A09	[67]
18	CGR6103	A08, D24	[68]
19	NAU0923	D09	[69]
20	NAU1043	A07	[56]
21	NAU1071	A03	[70]
22	NAU1093	A06	[70]
23	NAU1151	A12	[62]
24	NAU1190	A03	[70]
25	NAU1221	D05	[71]

No.	SSR marker	Chr. No.*	Reference
26	NAU1230	A05	[72]
27	NAU1255	D05	[62]
28	NAU2026	A05	[73]
29	NAU2300	A13	[62]
30	NAU2437	A01	[62]
31	NAU2508	D10	[62]
32	NAU7049	A10	[62]
33	BNL1231	A03	[73]
34	BNL2634	D07	[56]
35	BNL1167	A04	[65]
36	BNL1666	D01	[74]
37	CGR5866	D11	[75]
38	CIR0139	D05	[76]
39	CIR0246	D02	[77]
40	DPL0473	D02	[78]
41	DPL0590	A06	[79]
42	Gh262	A05	[78]
43	Gh433	A06	[80]
44	Gh591	D04	[81]
45	Gh592	A13	[76]
46	HAU0423	A11	[65]
47	HAU0878	A05	[81]
48	NAU0458	D01	[82]
49	NAU0837	A06	[56]
50	NAU0868	D03	[83]
51	CGR6078	A01	[84]

No.	SSR marker	Chr. No.*	Reference
52	DPL0131	D11	[66]
53	JESPR204	D13	[77]
54	NAU2443	D13	[85]
55	BNL0226	A03	[86]
56	BNL0530	D04	[86]
57	BNL1053	D11	[87]
58	BNL3424	D03	[62]
59	CGR5102	D08	[88]
60	CGR5597	A01	[89]
61	Gh034	A13	[82]
62	Gh056	D07	[72]
63	Gh110	D10	[82]
64	Gh132	D21	[90]
65	Gh243	A12	[90]
66	Gh247	A09	[65]
67	NAU2002	D07	[91]
68	NAU2265	A05	[92]
69	NAU2276	D09	[90]
70	NAU2277	A01	[21]
71	NAU2317	A10	[93]
72	NAU2336	D14	[94]

Table S2. Polymorphic SSR markers, and their polymorphism information content (PIC) and heterozygosity (He) values

Marker	Forward primer (5'-3')	Reverse primer (5'-3')	Molecular weight (bp)	PIC	He	Associated trait	Chr. No*	Reference
BNL0226	TTATTCTCACAGCCGGAACC	TTCACCCTCTCGCTTCTCAT	200, 230	0.353	0.459	Fiber quality	A03	[86]
BNL0530	CGTAGGATGGAAACGAAAGC	GCCACACTTTTCCCTCTCAA	120, 130, 180	0.374	0.498	Fiber quality	D04	[11,95]
BNL1053	AGGGTCTGTCATGGTTGGAG	CATGCATGCGTACGTGTGTA	190, 200	0,280	0,336	Fiber quality	D11	[87]
BNL1122	TCGATAACGGCTATAGTAATCTCTC	CAACAAATAAGCAGCCAAGAAA	170, 180	0.325	0.408	Fiber strength	D16, A07	[56]
BNL1231	TAATAAAAGGGAAAGGAAAGAGTT	TATGGCTCTAGAATATTCCCTCG	180, 190	0.324	0.408	Plant height, fruit branch number	A03	[73,96]
BNL1421	TGAAGATTTGGAGGCAATTG	GAAATCAAGCCTCAATTCGG	190, 210	0.370	0.490	Lint cotton yield, micronaire	A13	[57]
BNL1666	TGTCAGAAAAGTTTTTCCAAGG	AGATCATATTTAAAAGAAAAAGAAAACC	110, 120	0.324	0.408	Plant height, dry stem weight	A01	[74]
BNL1694	CGTTTGTTTTCGTGTAACAGG	TGGTGGATTCACATCCAAAG	210, 250	0.280	0.337	Seed index, fiber length	D16, A07	[57,58]
BNL2496	TCGAAATGAATTTAGATGACCA	TCCTTTTTTTTGTACTTCTCTTGC	95, 110	0.280	0.337	Aspartic Acid, Serine, Glycine, Alanine, Arginine, Proline	D17	[59]
BNL2634	AACAACATTGAAAGTCGGGG	CCCATCTTCTTATTTTTTTC	200, 230, 250, 280	0.369	0.489	Fiber length, fiber strength	D07	[56]
BNL3140	CACCATTGTGGCAACTGAGT	GGAAAAGGGAAAGCCATTGT	100, 110	0.325	0.408	Fiber strength	D23, A09	[56]
BNL3171	GAAAAATTGAGGAAGGACATACG	GGCCACAACCGAATTTACTG	100, 200	0.375	0.500	Fiber strength	D21	[60,97]
BNL3347	AGACTGACATGCAGCTTCCA	ATCTTAATTTTGAGTATAGGATAGGGG	100, 130	0.354	0.459	Hull percentage	A05	[61]
BNL3424	TGTGCCGTCTCAAAATGAAG	AAGACCAATCTGTTGCCAGC	160, 180	0,325	0,408	Relative malondialdehyde	D03	[62]
BNL3436	AACATAGCCTACCATTGCCG	TTGTTTGCCAAATTTGAAGC	190, 200, 500, 550, 800	0.338	0.431	Fiber length, fiber micronaire	D25	[56]
BNL3452	TGTAACTGAGCAGCCGTACG	GCCAAAGCAGAGTGAGATCC	170, 190	0.325	0.408	Relative malondialdehyde	D19, A05	[62]
BNL3601	TTCCGTTGATGGAAATTGAA	ACAAGAATGCGTGTGTCTGC	150, 180, 220, 320, 500, 700	0.375	0.500	Maturity, cell wall thickness	A05	[57]
BNL3792	TTCGAGATCCCCTGTTCTGA	CATATTCCAGTCAAACCAAACG	240, 550, 740, 1000, 1300	0.269	0.320	Relative plant height	A08	[62]
BNL3932	TCGGGATTTTTATGGCAAAG	GAACACTCAACAAGGGGGAA	240, 250	0.375	0.500	FOV resistance	A06, A02	[63]
CGR5597	ACATGGTGGGAATGAGAAGC	AATGTAGTGACGGGCCTTTG	170, 480	0,124	0,132	VW resistance	A01	[89]
CGR5602	ATCGCCATTGTTTACTGCTA	ACCCTTCCACCATTAAACCC	130, 150	0.325	0.408	Fiber length	A11	[65]
CGR5732	GCCAAGGTTCATTCCTGAAA	TATAGGGCTCATCAGGGTGG	140, 160	0.280	0.337	Fiber strength	D19, A05	[66]

Marker	Forward primer (5'-3')	Reverse primer (5'-3')	Molecular weight (bp)	PIC	He	Associated trait	Chr. No*	Reference
CGR5866	GCTTAGGTTGTCATCCTTATTCG	CCCTTTGTTCAATTTCTCGTG	100, 140	0.280	0.336	Fiber quality	D11	[75]
CGR6078	CATGCAAGAAAGCTGCTCAA	TAGGCATGTGTCTCCGTGTG	90, 110	0.324	0.408	Boll weight, fiber length, fiber strength, fiber elongation, fiber uniformity	A01	[84]
CGR6103	CAAAGGATGGGACACAGGTAA	TGCATTAGATACCGAAATGAGC	100, 120	0.325	0.408	Fiber length	A08, D24	[68]
CIR0139	AAACAAATGGAGAGGGT	ACCTGTGGTCTGCAAT	160, 180	0.280	0.336	Fiber quality	D05	[76]
CIR0246	TTAGGGTTTAGTTGAATGG	ATGAACACACGCACG	170, 200, 250, 300, 340, 400, 450	0.372	0.494	Salt tolerance	D02	[77]
DPL0131	ACATACGGGTTGAAATGTACTCCT	ATGAATGCAGATCATTACGCCT	190, 210	0.324	0.408	Fiber elongation, fiber strength,	D11	[66]
DPL0473	CGTTACAGGCGTAACTAAAGAGGT	ATGTATATGACATTGAGTGGCTGC	180, 200	0.324	0.408	FOV resistance	D02	[78]
DPL0590	GATTTACTTTAAGGAGGCGAAAC	AAAGGTACACTCATGCGACTGAC	220, 250	0.280	0.336	Fiber quality	A06	[79]
Gh056	TCCATTAGACAAAGTTTTCTAAAGTTC	TGAGACTTCCAACCAGATACAG	80, 110	0.324	0.408	Fiber quality	D07	[72]
Gh110	ACCATCCCAAAGAATCATCCTC	ACTAAAACCAAGGCAATAAAGTG	180, 190	0.280	0.336	Fiber quality	D10	[82]
Gh243	CAGAAGGTTATGCAAACAACATGCA	CTAAACTCTCTCTGCTGTGTTCC	60, 150, 160	0,369	0,489	FOV resistance	A12	[90]
Gh247	CTTCTTCCGCCACGTAAGTCC	CAGCCTAACCAAGAACCCAATCG	115, 160	0,517	0,594	Fiber micronaire	A09	[65]
Gh262	GTCAACAACCTTAAAATTGCCATAGGT	TGGACTACCAATTAACTTGCCACG	150, 185, 200	0.374	0.498	FOV resistance	A05	[78]
Gh433	TACCACATTGGATGTTTGCAAACCC	ATAGCAAACTGGAATCACTCCAAGC	115, 170, 185	0.304	0.375	VW resistance	A06	[35]
Gh592	TTGTTATCTAACTTCTGTTACTCCTAAC	TTGTTTAGCTCTTCTATACTTGAATTCC	50, 100, 110	0.324	0.408	Fiber quality	A13	[76]
HAU0423	CGATGGAAGAAAAATCGAAA	ATATTTCGACCAAGGAGTCG	170, 190	0.324	0.408	VW resistance	A11	[65]
HAU0878	TCATTCCTGAAACCCAAAAT	CTAACAGGGGTGACATAGGG	120, 140	0.280	0.336	Fiber quality	A05	[81]
JESPR204	CTCCAGGTTCAATGGTCTG	GCCATGTTGGACAAGTAGTC	160, 190	0.324	0.408	Seedling period, bud period, flower and boll period, salt tolerance	D13	[77]
NAU0458	AGGACTTGTCCACGTGCTTC	TTTGATTCTTTTCGGCTGCT	140, 195, 280, 500, 680	0.369	0.489	Salt tolerance	D01	[98,99]
NAU0868	GGCAAAACCATAAGGGTAAC	TAGCGTGAGATTGTGGCTTA	190, 210	0.280	0.336	Fiber quality	D03	[83]
NAU0923	GGAATTCAAGGTTGAAGGAG	GGAATTCAAGGTTGAAGGAG	210, 230	0.325	0.408	Fiber quality	D09	[69]
NAU1043	GTATCCGCCCACAAATAAAG	GCATCGTGAGAGAAAGTGAA	220, 290	0.280	0.336	Fiber quality	A07	[56]

Marker	Forward primer (5'-3')	Reverse primer (5'-3')	Molecular weight (bp)	PIC	He	Associated trait	Chr. No*	Reference
NAU1093	AAATGGCGTGCTTGAAATAC	TGTGATGAAGAACCCTCTCA	150, 190	0.353	0.459	VW resistance	A06	[70]
NAU1151	TGGTTGCTTTGTATTGCTTG	CGTACTTGCGAAAGAGAACA	100, 200	0.375	0.50	Relative malondialdehyde	A12	[62]
NAU1190	CCATGTCCGTATCCATGTTA	TAAGGCAAGATAGGGTCAGG	220, 250, 280	0.369	0.489	Fiber quality	A03	[70]
NAU1221	CATGCAAATCCATGCTAGAG	AGGTTTCTTTGGTGGTGAAA	240, 260	0.324	0.408	FOV resistance	D05	[71]
NAU1230	CATGCAAATCCATGCTAGAG	TCAAAAGGTTCTTTGGTGGT	230, 250	0.324	0.408	Fiber quality	A05	[72]
NAU1255/ NAU1042	CATGCAAATCCATGCTAGAG	GGTTTCTTTGGTGGTGAAAC	230, 250	0.324	0.408	Relative root dry matter	D05	[62]
NAU2002	GCCCTTTTTGGTAGATGAAC	ATCACTTCAGCTGGGGTTT	420, 470	0,324	0,408	Fiber strength	D07	[91]
NAU2026	GAATCTCGAAAACCCCATCT	ATTTGGAAGCGAAGTACCAG	180, 200, 210	0.296	0.362	Plant height	A05	[73]
NAU2277	GAACTAGCCACATGATGCAC	TTGTTGAGGCATTAGTTTGC	120, 140	0,551	0,622	Drought tolerance	A01	[21]
NAU2317	GACTCCAGCCTTCACACAT	TGGAAGAGTATAACGGCAGA	180, 190	0,475	0,545	Fiber quality	A10	[93]
NAU2336	TGGAAAAGGAAGAGGAGAGA	CCCTGAAGTTGTCAAGCTCT	140, 150, 160, 180	0,516	0,581	Biotic factors, cotton leaf curl disease, seed cotton yield per plant	D02	[94]
NAU2443	CGTTGAGAAGGAAAGCCTAA	AGCCTGCTTCATGTTCTTTT	100, 120, 140, 170, 190	0.296	0.362	Fiber strength, fiber elongation, fiber uniformity height of first fruiting branch node	D13	[85]
NAU7049	AGGTACCTCTCCTGACTCT	AATCTTCTTGAAATCGAAC	250, 350	0.280	0.336	Relative superoxide dismutase	A10	[62]
